# Small fluorescent molecules for monitoring autophagic flux

**DOI:** 10.1002/1873-3468.12979

**Published:** 2018-02-02

**Authors:** Hidefumi Iwashita, Hajime Tajima Sakurai, Noriyoshi Nagahora, Munetaka Ishiyama, Kosei Shioji, Kazumi Sasamoto, Kentaro Okuma, Shigeomi Shimizu, Yuichiro Ueno

**Affiliations:** ^1^ Dojindo Laboratories Kumamoto Japan; ^2^ Department of Chemistry Faculty of Science Fukuoka University Japan; ^3^ Department of Pathological Cell Biology Medical Research Institute Tokyo Medical and Dental University Japan

**Keywords:** autolysosome, autophagosome, autophagy, fluorescent probe

## Abstract

We have developed two types of fluorescent probes, DALGreen and DAPGreen, for monitoring autophagy, that exhibit fluorescence upon being incorporated into autophagosomes. DALGreen enhances its fluorescence at acidic pH, which is favorable for monitoring late‐phase autophagy, whereas DAPGreen remains fluorescent with almost constant brightness during the autophagic process. With these probes that stain autophagosomes as they are being formed, the real‐time change of autophagic phenomena of live cells may be traced, which is an advantage over conventional approaches with small molecules that stain mature autophagosomes. The use of both dyes allows monitoring of the membrane dynamics of autophagy in any type of cell without the need for genetic engineering, and therefore, will be useful as a tool to study autophagic phenomena.

## Abbreviations

**LC3**, microtubule‐associated protein 1 with light chain 3

**MEF**, mouse embryonic fibroblast

**PeT**, photoinduced electron transfer

Autophagy is intracellular degradation process for misfolded or aggregated proteins, dysfunctional organelles, and foreign pathogens, through which they are recycled or metabolized 1, 2, 3, 4. During the process, these cellular components are sequestered by a double‐membrane vesicle, called autophagosome, which is then fused with lysosome for enzymatic hydrolyses in the acidic environment. Being cell's vital strategy for survival, its impaired function is associated with cancer, inflammatory, as well as neurodegenerative diseases. To understand the expanding roles of autophagic phenomena and further explore its biology, development of a sensitive and reliable detection technique with living cells is needed, considering the limited availability of electron microscopy which is most reliable in identifying autophagy. Anti‐LC3 antibody is the most widely used biological marker to detect autophagy by western blot analysis and/or immunostaining analysis. LC3‐I, a cytosolic form of LC3, is conjugated with phosphatidylethanolamine to form LC3‐II, and LC3‐II is recruited to autophagosomal membrane, which is an important indication of autophagic activity 5. LC3 tagged with green fluorescent protein (GFP‐LC3) has been also used widely for live‐cell imaging of autophagy, in which the GFP fluorescence is diminished when autophagosomal pH drops in the degradation phase. Therefore, GFP‐LC3 is a specific marker for early‐phase autophagy although it tends to aggregate when overexpressed 6.

Fluorescent small molecules for visualizing autophagosomes have been developed to monitor autophagy without genetic engineering and allowed easy detections. They have helped neighboring research fields to focus on relations to autophagy. Monodansylcadaverine (MDC) has served as a conventional small‐molecule fluorescent probe for autophagic vacuoles, but its low specificity as well as the cytotoxicity is the limiting factor in addition to photo‐bleaching due to the ultraviolet excitation wavelength 7. CYTO‐ID dye has been recently developed and commercialized as a kit product in an effort to overcome the limitations of MDC; it was reported to label autophagic compartments with minimal staining of lysosome and allow monitoring of the whole activity of autophagy (autophagic flux) in combination with inhibitors 8. However, the labeling mechanism as well as its chemical structure has not been disclosed except that the dye is known to be some kind of cationic amphiphilic dye, and the lack of information could narrow the access of this kit to biomedical research community. Jiang *et al*. 9 recently reported a two‐photon fluorescent probe, Lyso‐OC, for real‐time monitoring of autophagy as a fluorescence decrease by detecting the change in the lysosomal polarity during the process, but it requires special instrumental settings to monitor, which may also narrow the applicability. These works and their limitations prompted us to develop small‐molecule fluorescent probes that are suitable for widely used fluorescence microscopy and can be used to monitor autophagic flux with any types of cells.

We have reported in our previous study a fluorescent probe for visualizing mitophagy, mitochondria‐selective autophagy, which senses mitochondrial acidification through a photoinduced electron transfer (PeT) mechanism 10. Using a similar approach, we developed in this study two types of small fluorescent molecules, DALGreen and DAPGreen (Fig. S1), that are able to stain autophagosomes with fluorescence enhancement in the hydrophobic environment. The fluorescence of DALGreen enhances at acidic pH, which is suitable for monitoring the degradation phase of autophagy. DAPGreen, on the other hand, has a pH‐independent fluorescence profile and remains fluorescent with almost constant intensity throughout the process of autophagy. We report here live‐cell imaging of autophagosomes stained with these dyes *ex vivo* in details.

## Materials and methods

### Reagents and instruments

All chemicals were purchased from Tokyo Chemical Industries (Tokyo, Japan), Wako Pure Chemical Industries (Osaka, Japan), Sigma‐Aldrich (St, Louis, MO, USA), and Thermo Fisher Scientific (Waltham, MA, USA), and were used as purchased. LysoTracker Deep Red (Thermo) and LysoTracker Red DND‐99 (Thermo) were used for staining the lysosomes following the product manual. ^1^H‐NMR and ^13^C‐NMR spectra were recorded on a Bruker AVANCE III HD 400 MHz spectrometer. Mass spectra were measured with a JMS‐T100CS (JEOL, Tokyo, Japan) or a Waters SQD2 (Waters, Milford, MA, USA) mass spectrometer. UV‐visible spectra were obtained on a UV‐2450 UV/Vis spectrophotometer (SHIMADZU, Kyoto, Japan) and fluorescence spectroscopic studies were performed on a FP‐6300 fluorescence spectrophotometer (JASCO, Tokyo, Japan). Fluorescence images were obtained with LSM710 and LSM800 confocal laser scanning microscopies (ZEISS, Oberkochen, Germany). Cell viability assays on 96‐well plates were performed with an Infinite 200 pro plate reader (TECAN, Maennedorf, Switzerland).

### Cell culture

The HeLa and mouse embryonic fibroblast (MEF) cells were cultured in Minimum Essential Media (MEM, Thermo) or Dulbecco's modified Eagle's medium (DMEM) (high glucose, Nacalai, Kyoto, Japan) supplemented with 10% (v/v) fetal bovine serum, 2 mm l‐glutamine, 1% nonessential amino acids, 100 unit·mL^−1^ penicillin, and 100 μg·mL^−1^ streptomycin in a humidified 5% or 10% CO_2_ incubator at 37 °C. ULK1/ULK2 DKO MEFs were kindly provided by Professor Tooze (London Research Institute). For staining with DALGreen and DAPGreen, seeded cells were preincubated with these dyes in the culture medium for 30 min at 37 °C, then washed with the culture medium and stimulated to induce autophagy. The fluorescence images were taken in the same conditions (laser power and detector gains). For inducing autophagy, the cells were cultured with the amino acid‐deprived DMEM (Wako) to stimulate starvation or cultured with 500 nm rapamaycin‐containing medium. Chloroquine (10 μm), bafilomycin A1 (0.1 μm), and 3‐MA (5 mm) were used to inhibit autophagy.

### DNA transfection

To transiently express tagRFP‐LC3 and Lamp1‐tagRFP, each plasmid was transfected into MEF and HeLa cells using the Neon transfection system (Thermo) and Lipofectamine 2000 (Thermo). Both expressing plasmids were modified from the plasmids used in the previous study to the pcDNA3.1 tagged with tagRFP backbones 4.

### Cell viability assay

Cell viabilities were measured with CCK‐8 (Dojindo Laboratories, Kumamoto, Japan) following the manufacturer's manual. HeLa cells were seeded on a 96‐well plate and the plate was incubated with the culture medium at 37 °C in a 5% CO_2_ incubator for 24 h. The medium was replaced with the culture medium containing various concentrations of DALGreen or DAPGreen (0.1, 0.5, and 1.0 μm); the cells were further incubated for 30 min at 37 °C, washed with the culture medium, and incubated for another 24 h. After CCK‐8 was added to each well, the cells were incubated for another 2 h. The absorbance at 450 nm of each well was measured with a microplate reader.

### Western blot analysis

Cells were lysed in a sample buffer (50 mm Tris/HCl at pH 6.8, 5% glycerol, and 1% SDS) and boiled for 3 min at 95 °C. The samples were separated on SDS/PAGE, transferred onto a poly(vinylidene difluoride) membrane (Trans‐Blot Turbo, BIO‐RAD), and detected with a rabbit anti‐LC3 antibody (PD014, MBL) and a mouse anti‐actin antibody (MAB1501, Millipore). Horseradish peroxidase‐conjugated antibodies (Santa Cruz Biotechnology, Dallas, TX, USA) were used as secondary antibodies (at 1 ∶ 20 000 dilution). Chemiluminescent signals detecting with ECL Western Blotting Substrate (Thermo) were scanned by a Multi‐Imager (Gellex, Tokyo, Japan).

### Immunocytochemical analysis

Autophagy‐induced cells were fixed in 4% paraformaldehyde in phosphate‐buffered saline (PBS) for 30 min at room temperature, permeabilized with 0.1% Triton‐X100 in PBS, blocked with 20% Blocking One (Nacalai Tesque), and then incubated with a diluted rabbit anti‐LC3 antibody (PD036, MBL) in 20% Blocking One in PBST (0.1% Tween‐20 in PBS) for 16 h at 4 °C. After being washed with PBST, cells were incubated with Alexa 488‐conjugated secondary antibody (Thermo) and DAPI (Dojindo Laboratories) in PBST for 2 h.

### Liposome assay

Liposome was prepared by a hydration method for giant liposomes using Egg York Phosphatidylcholine (DYPC). About 1 mg of DYPC was dissolved in CHCl_3_, and the organic layer was evaporated by N_2_ purge. To the residue was added prewarmed water (80 °C) with or without DALGreen, DAPGreen or **3g**, which was incubated for 1 h in water bath at 80 °C. The resulting mixture was centrifuged by a microcentrifuge and the precipitate was washed with water twice. The obtained liposomes were measured with a confocal fluorescent microscopy. Fluorescence images for these dyes were obtained as described above.

## Results and Discussion

In designing a molecule that has an affinity to autophagosomal membrane and increases its fluorescence upon binding, we first selected 1,8‐naphthalimide as a fluorogenic scaffold, which was expected to enhance the fluorescence in a hydrophobic environment such as double‐membrane space of an autophagosome. We envisioned that introduction of an alkyl group of different length (‘tail’ group in Fig. S1) to the dicarboximide moiety further increases the hydrophobicity of this molecule, and also that incorporation of a piperazine group (‘head’ group in Fig. S1) to the other side of the molecules makes the fluorescence pH‐dependent. The syntheses of **4a**‐**d** that we designed depending on the alkyl chain length were carried out as outlined in Scheme S1, starting from commercially available 4‐bromo‐1,8‐naphthalic anhydride in four steps in a moderate overall yield. Compounds **4e** and **4f,** having an amino group at the tail end, were also synthesized in a similar manner.

First, to clarify the effect of the alkyl chain length as well as the amino group at the tail end, we compared **4a‐f** in their fluorescence enhancement using HeLa cells under nutrient‐deprived conditions (Fig. S2). While only DALGreen (**4b**) and **4c** showed the fluorescence in starved cells, DALGreen that has a medium‐length pentyl group was found to give the strongest signal and hence was used in the following experiments. The observation that less hydrophobic (**4a**) and more hydrophobic (**4c** and **4d**) compounds exhibited very weak fluorescence implies that another factor may be important to regulate an interaction between the dyes and the autophagosomal membranes. Although the reason is unclear, the amino group at the tail end of **4e** and **4f** showed no significant effects in the fluorescence enhancement.

The fluorescence property of DALGreen under cell‐free conditions at various pH is shown in Fig. S3. When pH was decreased from 8 to 4, the fluorescence enhanced about 100‐fold, whereas the absorption maximum of DALGreen slightly shifted to shorter wavelength (Fig. S3A and S3B). The fluorescence enhancement of DALGreen at acidic pH may be explained by a PeT mechanism in which the piperazine group quenches the fluorescence at neutral pH and the fluorescence is restored owing to the protonation of this quenching group at acidic pH. Moreover, the fluorescence of DALGreen was largely influenced by hydrophobicity, as observed in the decreasing fluorescence intensity in aqueous acetonitrile in proportion with increasing amount of water (Fig. S3C).

**Figure 1:**
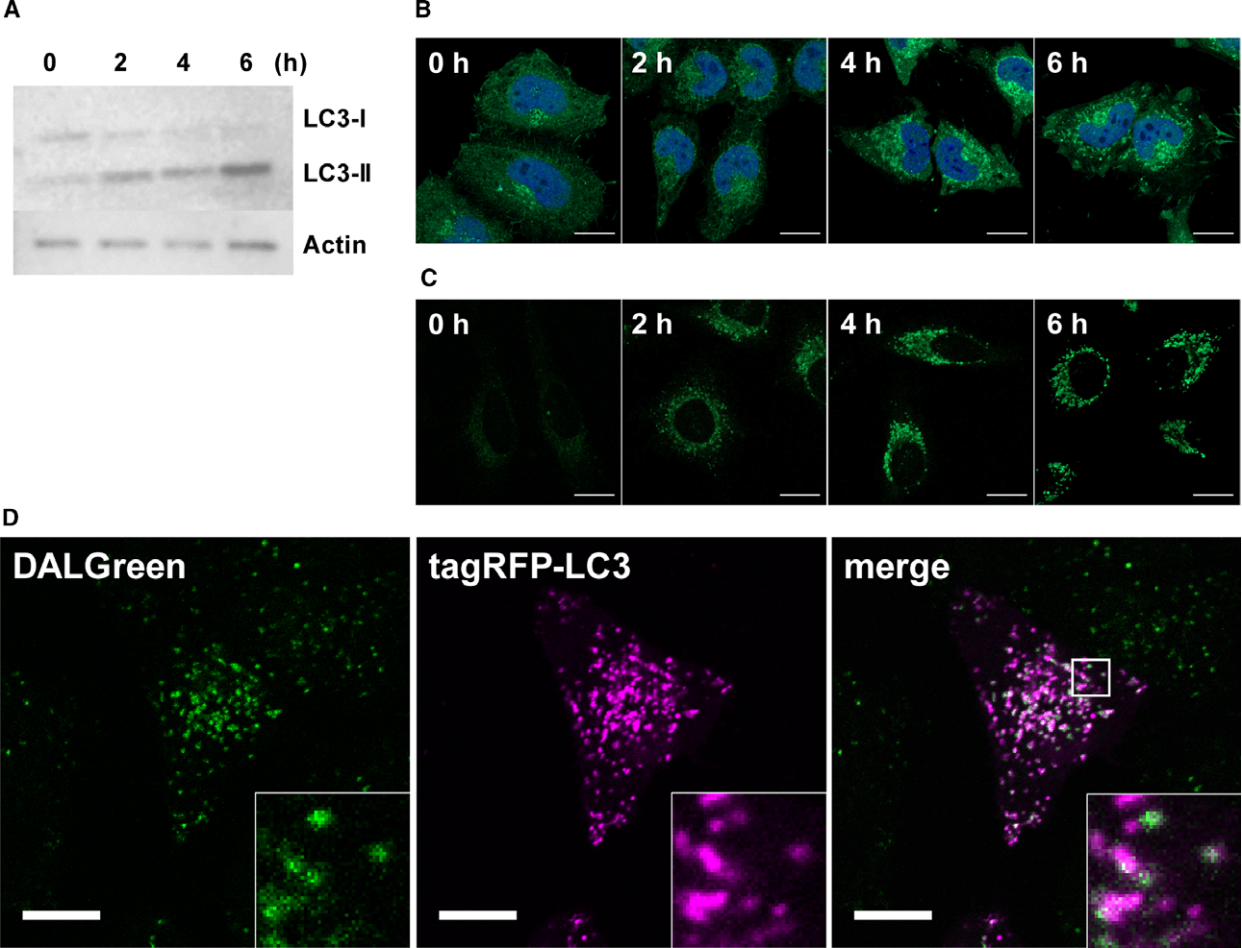
(A–C) HeLa cells stimulating by nutrient‐deprivation were analyzed in every 2 h. Bars = 20 μm. (A) Relative expression level of LC3‐I and LC3‐II were detected using with anti‐LC3 antibody and anti‐actin antibody for loading control of western blotting analysis. (B) Immunocytochemical analysis with anti‐LC3 antibodies (Green) and DAPI (Blue). (C) Live‐cell imaging with 1 μm DALGreen staining. (D) Live‐cell imaging of 4 h rapamycin treated HeLa cells colabeling with 1 μm DALGreen and tagRFP‐LC3. Bars = 10 μm.

We next investigated DALGreen responses to autophagy in cultured cells. LC3‐II, an autophagosomal membrane marker, was converted from LC3‐I in association with progress of autophagic process (Fig. 1A). In accord with this, endogenous LC3 forms puncta corresponding to the increase of DALGreen intensity level (Fig. 1B,C). To validate the correspondence between the DALGreen fluorescence and the autophagic activity, immunocytochemistry and western blot analysis were performed using an anti‐LC3 antibody in starved HeLa cells (Fig. 1A,B). These fluorescence images appear to indicate that fluorescent DALGreen stains some autophagic components, most likely autophagosomal membranes, which are degraded in an acidic environment. Then, to confirm this hypothesis, we carried out live‐cell imaging in a human cell line (HeLa) and in a mouse cell line (MEF), expressing red fluorescence tagged LC3 (Figs 1D and S4A). As the fluorescence of tagRFP is pH‐insensitive, tagRFP‐LC3 is able to label not only autophagosomes but autolysosomes. Therefore, almost all of DALGreen signals were colocalized with LC3 reasonably, indicating that DALGreen stains autolysosomes that are the unique acidic compartment in autophagy. It was also supported by the result of colocalization analysis with the lysosomal membrane marker, Lamp1‐tagRFP (Fig. S4B).

**Figure 2:**
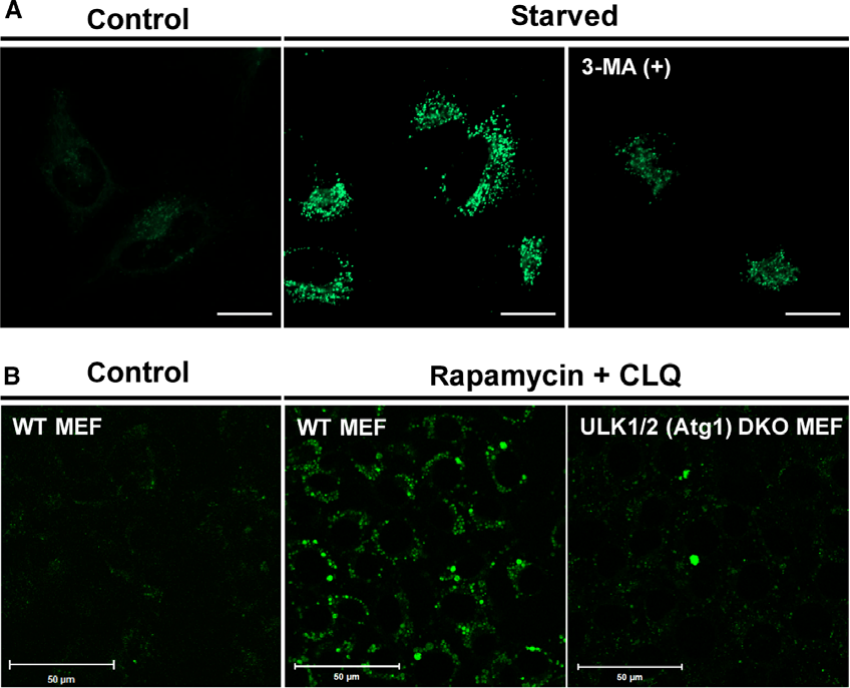
(A, B) Live‐cell imaging with 1 μm DALGreen staining. (A) About 5‐h starved HeLa cells were treated with or without 3‐MA. Bars = 20 μm. (B) Wild‐type and ULK1/ULK2 DKO MEF cells treated with or without rapamycin and CLQ for 8 h. Bars = 50 μm.

Furthermore, we observed the DALGreen fluorescence in comparing with inhibited conditions to confirm the property of DALGreen as an authentic autophagy dye. First, we treated the cells with 3‐methyladenine (3‐MA) that inhibits phosphatidylinositol 3‐kinase activity and autophagosome formation in the initial phase of autophagy, and found that the inhibitor efficiently quenched the DALGreen fluorescence induced by starvation in HeLa cells 11 (Fig. 2A). Similarly, we next examined the DALGreen fluorescence in ULK1/ULK2 double‐knockout (DKO) MEF cell, which is defective for the homologous functions of yeast Atg1 protein 12. As expected, the defects dramatically decreased DALGreen staining even in the presence of chloroquine (CLQ), which enhances the accumulation of the DALGreen fluorescence by inhibiting the lysosomal functions as described below (Fig. 2B). These results indicated that the intensity of DALGreen staining reflects the activity of autophagy. Our further observation in wild‐type and ULK1/ULK2 DKO MEFs costained with DALGreen and LysoTracker, a labeling reagent for acidic organelles, revealed that a portion of lysosomes were colocalized with DALGreen signals (Fig. S5). These results implied that DALGreen selectively stains autolysosomes as distinct from another lysosome such as primary lysosomes. Together, DALGreen is incorporated into the autophagic component as the nonprotonated form (with quenched fluorescence), and then the membranes are transported into autolysosomes in accord with potentiating the DALGreen fluorescence. Correspondingly, when the dye was added after the induction of autophagy, it did not produce any fluorescence, but the intensity increased following the induction of autophagy (Fig. S6). This result clearly indicated that DALGreen is not directly staining autophagosomes; DALGreen, thereby, stains only newly synthesized autophagosomes. This is an outstanding feature of DALGreen compared with other dyes, such as CYTO‐ID that gathers both signals from autophagosomes and autolysosomes.

As DALGreen has a pH‐dependent fluorescence profile, we next focused on developing pH‐independent fluorescent probes to visualize the early‐phase autophagosomes. We synthesized analogous compounds, DAPGreen (**6a**) and **6b**, in the same manner for **4a‐f** as shown in Scheme S2. DAPGreen has a similar molecular size to DALGreen and has 5‐aminopentyl moiety as a head group instead of the pH‐sensitive piperazine moiety of DALGreen. To affirm the importance of PeT effect as seen in DALGreen, we designed **6b** with an additional amino functionality in the head group (Fig. S1; X). As expected, the fluorescence of DAPGreen was found to be pH‐independent and showed a remarkable stability in pH sensitivity in contrast to **6b** (Fig. S7B *versus* S7D), even though the absorption spectra indicated a slight pH‐dependency (Fig. S7A *versus* S7C). The difference in the pH sensitivity of the fluorescence between DAPGreen and **6b** results from the presence of nitrogen in the center of the head group, such as in DALGreen, suggesting that the PeT effect is caused by the nitrogen not conjugated with the naphthalene ring.

**Figure 3:**
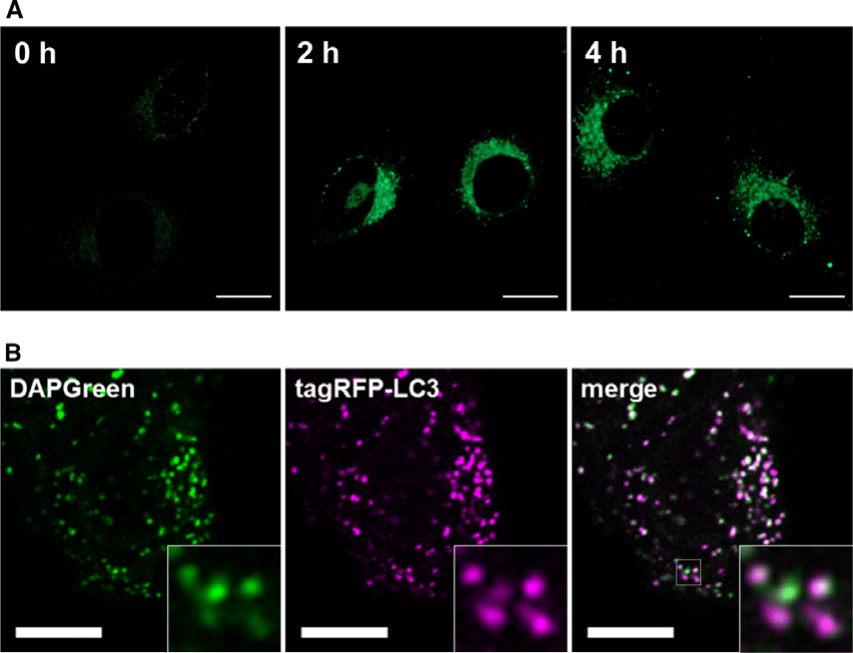
(A) Time lapse imaging of starved HeLa cells stained with 0.1 μm DAPGreen in every 2 h. Bars = 20 μm. (B) Fluorescent images of 4 h rapamycin treated HeLa cell colabeling with 0.5 μm DAPGreen and tagRFP‐LC3. Bars = 10 μm.

To confirm the staining profile of DAPGreen in cultured cells, we then carried out a series of observations in a similar way as for DALGreen. DAPGreen signals were also increased after inducing autophagy and were double‐labeled with the tagRFP‐LC3‐positive autophagosomes (Figs 3 and S8A). Fortunately, the brightness of DAPGreen fluorescence was stronger than that of DALGreen implying that DAPGreen stains much more autophagosomal membranes than does DALGreen as expected (Figs 1C and 3A). In colocalization experiments, the staining of DAPGreen also showed the higher correlation with tagRFP‐LC3 signals than that of DALGreen (Fig. 3B and S8A *versus* Fig. 1D). On the other hand, colabeling with Lamp1‐tagRFP demonstrated that a number of DAPGreen signals were isolated from lysosomes, while the other DAPGreen signals were well colocalized with Lamp1‐tagRFP (Fig. S8B). These results indicate that DAPGreen stains not only the late‐phase autolysosomes but also the early‐phase autophagosomes.

**Figure 4:**
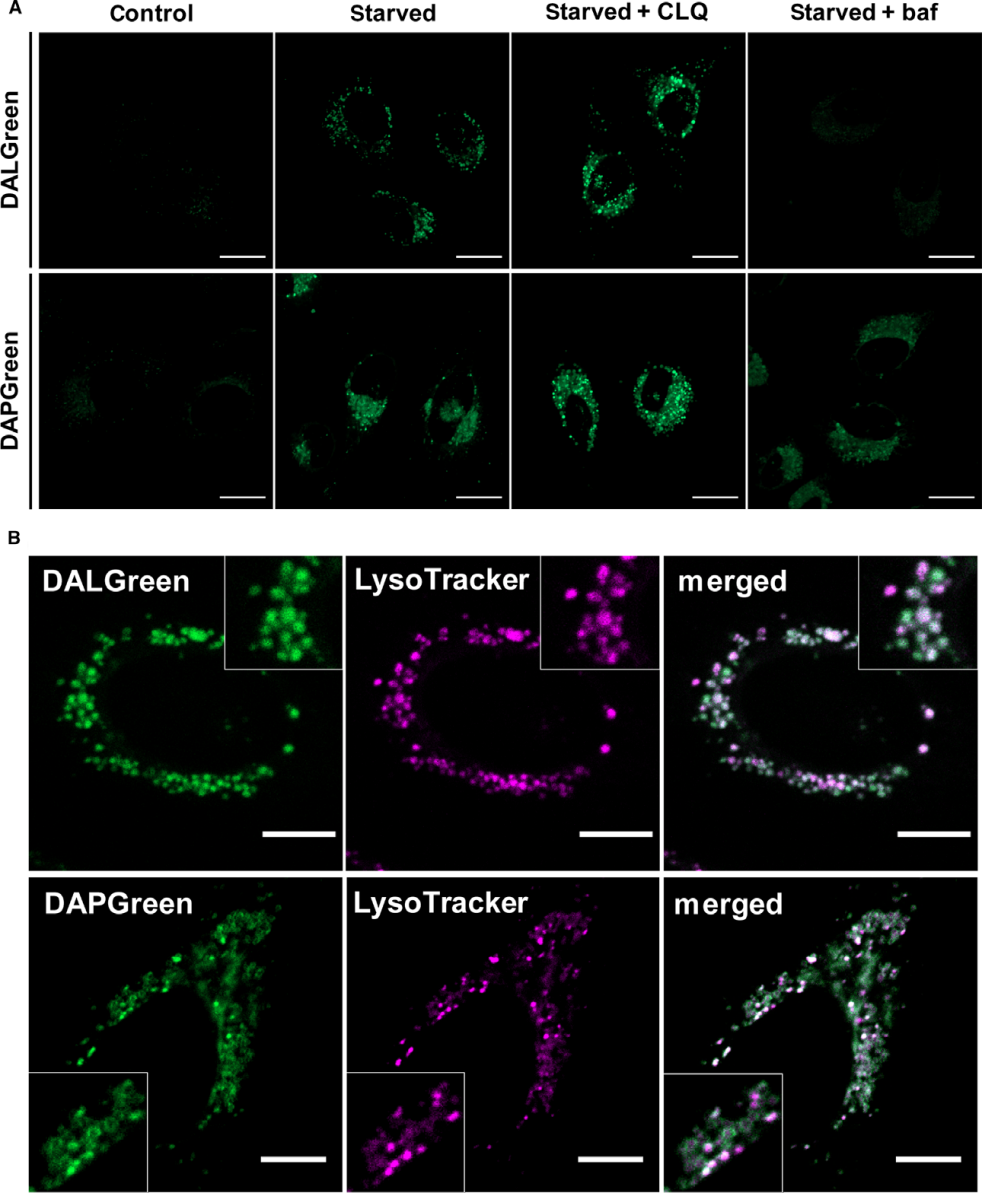
(A) Live‐cell imaging of 5‐h starved HeLa cells staining with 1 μm DALGreen or 0.1 μm DAPGreen in the presence or absence of 10 μm CLQ or 0.1 μm bafilomycin A1. Bars = 20 μm. (B) Live‐cell imaging of 3 h starved HeLa cells costaining with 50 nm LysoTracker Deep Red and 1 μm DALGreen or 0.1 μm DAPGreen. Bars = 20 μm.

We next focused on the difference between the profiles of DALGreen and DAPGreen in details. Chloroquine (CLQ) and bafilomycin A1 are typical autophagy inhibitors, such as 3‐MA, which was used in Fig. 2B for inhibiting early‐phase autophagy, and prevent late‐phase autophagy by the different mechanisms. CLQ is thought to inhibit a degradation activity in autolysosomes, even though its target is unclear 13, whereas bafilomycin A1 prevents fusion within autophagosomes and lysosomes by blocking the autophagosomal acidification via vacuolar H^+^ ATPase activity 14, 15. CLQ inhibition made the intensity of both dyes brighter suggesting that the swelled autolysosomes are fully filled with autophagic components by degradation retardation and still maintain their inside pH as acidic (Fig. 4; starved + CLQ). This hypothesis may explain the efficient enhancement of the DALGreen fluorescence by CLQ treatment. Bafilomycin A1 quenched the fluorescence of DALGreen completely because this inhibitor blocks pumping in H^+^ and maintains the autophagosomal pH as neutral (Fig. 4; starved + baf). By contrast, DAPGreen seemed to be less responsive to bafilomycin A1 suggesting that the fluorescence intensity is constant because of the absence of pH‐sensitive nitrogen atom in the head group. Through these observations, we found the characteristic staining pattern in DAPGreen. The magnified images revealed that a portion of DAPGreen fluorescence shows membrane‐like structures as seen with ‘ring’ signals, while the DALGreen fluorescence never forms ‘ring’ signals (Fig. 4B). This may be because the autophagosomal membranes stained by DALGreen are already degraded in autolysosomes. Therefore, these results also favor the molecular mechanisms of staining by these probes. Finally, we investigated the cytotoxicity of DALGreen and DAPGreen and found that both dyes were not cytotoxic at least up to 1.0 μm, the concentration we used in these live‐cell experiments (Fig. S9).

As we were interested to know whether the terminal amino group of DALGreen and DAPGreen is necessary for the incorporation of these dyes into autophagosomal membrane, we synthesized **3g** as outlined in Scheme S1. This compound does not have terminal amino functionalities either in the head or in the tail group (Fig. S1). We performed staining artificial liposomes and HeLa cells with **3g** and detected none of fluorescence, indicating that **3g** is involved neither in the liposomal nor autophagosomal membrane formation, in contrast to DALGreen and DAPGreen (Fig. S10 and S11). These findings led us to assume that these amphiphilic dyes of a detergent‐like structure with a terminal amino functionality in the head group mimic intramembrane phospholipids such as phosphatidylethanolamine, and that the most part of the dye structure is embedded in the hydrophobic membrane space (Fig. S12). This staining mechanism was also supported by an experiment with fixed HeLa cells stained by DALGreen and DAPGreen, in which the fixed cells were permeabilized by Triton x‐100. Both experiments resulted in a significant reduction of the fluorescence intensity probably because the dyes leak out from the membranes (data not shown). Together, it is most likely that DALGreen and DAPGreen are taken up into the autophagic membranes.

In conclusion, much remains to be elucidated in the research field of autophagy, and there is need for reliable and convenient methods for monitoring autophagy. By a chemical biology approach, we have developed two small‐molecule fluorescent probes, DALGreen and DAPGreen, for monitoring autophagy. DALGreen is suitable for monitoring the autophagic flux and DAPGreen has an ability to stain the whole process of autophagy. In future, these probes will be valuable for studying the molecular mechanisms and physiological roles of autophagy and provide novel approaches without the need for genetic engineering, particularly in histological analyses.

## Conflict of interest

Hidefumi Iwashita, Munetaka Ishiyama, Kazumi Sasamoto and Yuichiro Ueno are employees of Dojindo Laboratories, manufacturer of DALgreen.

## Supporting information


**Fig. S1.** Chemical structures of DALGreen, DAPGreen, and their analogous compounds.
**Fig. S2.** Fluorescence images of DALGreen and its analogs (1 μm) with HeLa cells under nutrient‐rich (control) or nutrient‐deprived condition (starved, 5 h). Scale bar = 10 μm.
**Fig. S3.** Absorption (A) and emission (B) spectra of DALGreen (5.0 μm) in buffer solutions (pH 4.0–8.0), excited at 405 nm. (C) Fluorescence spectra of DALGreen (5.0 μm) excited at 405 nm in aqueous acetonitrile (ACN) solutions. A working solution of DALGreen (1.0 mm in DMSO) was diluted with MES buffer.
**Fig. S4.** Confocal microscopic images of DALGreen (1.0 μm).
**Fig. S5.** Confocal microscopic images of wild‐type (upper panel) and ULK1/2 double‐knockout MEF cells (bottom panel), costained with DALGreen (1.0 μm) and LysoTracker (0.1 μm).
**Fig. S6.** Reversed staining procedure with DALGreen.
**Fig. S7.** Absorption and emission spectra of DAPGreen (A and B, respectively) and **6b** (C and D, respectively), excited at 450 nm in buffer solutions (pH 4.0–8.0).
**Fig. S8.** Confocal microscopic images of DAPGreen (0.1 μm).
**Fig. S9.** Cell viabilities of DALGreen and DAPGreen for HeLa cells measured by CCK‐8.
**Fig. S10.** Confocal microscopic images of liposomes treated with DALGreen, DAPGreen, or **3 g** in the formation of double membrane.
**Fig. S11.** Live‐cell imaging of starved HeLa cells stained with DALGreen or **3 g** for 5 h.
**Fig. S12.** A proposed staining mechanism of autophagosomal membrane with DALGreen or DAPGreen.
**Scheme S1.** Synthesis of DALGreen (**4b**) and its analogous compounds.
**Scheme S2.** Synthesis of DAPGreen (**6a**) and **6b**.
**Appendix S1.** Syntheses of DALGreen and DAPGreen.
