# Supplementary material for: Small fluorescent molecules for monitoring autophagic flux
Source: FEBS Lett. 2018 Feb 2;592(4):559–67. doi: 10.1002/1873-3468.12979 (PMC5947577; doi:10.1002/1873-3468.12979)
Supplement: Supplementary file 1 — Fig. S1. Chemical structures of DALGreen, DAPGreen, and their analogous compounds. Fig. S2. Fluorescence images of DALGreen and its analogs (1 μm) with HeLa cells under nutrient‐rich (control) or nutrient‐deprived condition (starved, 5 h). Scale bar = 10 μm. Fig. S3. Absorption (A) and emission (B) spectra of DALGreen (5.0 μm) in buffer solutions (pH 4.0–8.0), excited at 405 nm. (C) Fluorescence spectra of DALGreen (5.0 μm) excited at 405 nm in aqueous acetonitrile (ACN) solutions. A working solution of DALGreen (1.0 mm in DMSO) was diluted with MES buffer. Fig. S4. Confocal microscopic images of DALGreen (1.0 μm). Fig. S5. Confocal microscopic images of wild‐type (upper panel) and ULK1/2 double‐knockout MEF cells (bottom panel), costained with DALGreen (1.0 μm) and LysoTracker (0.1 μm). Fig. S6. Reversed staining procedure with DALGreen. Fig. S7. Absorption and emission spectra of DAPGreen (A and B, respectively) and 6b (C and D, respectively), excited at 450 nm in buffer solutions (pH 4.0–8.0). Fig. S8. Confocal microscopic images of DAPGreen (0.1 μm). Fig. S9. Cell viabilities of DALGreen and DAPGreen for HeLa cells measured by CCK‐8. Fig. S10. Confocal microscopic images of liposomes treated with DALGreen, DAPGreen, or 3 g in the formation of double membrane. Fig. S11. Live‐cell imaging of starved HeLa cells stained with DALGreen or 3 g for 5 h. Fig. S12. A proposed staining mechanism of autophagosomal membrane with DALGreen or DAPGreen. Scheme S1. Synthesis of DALGreen (4b) and its analogous compounds. Scheme S2. Synthesis of DAPGreen (6a) and 6b. Appendix S1. Syntheses of DALGreen and DAPGreen. [file FEB2-592-559-s001.pdf]

Supporting information

Fluorescent small molecules for monitoring autophagic flux

Hidefumi Iwashita*[,1,2], Hajime Tajima Sakurai[3], Noriyoshi Nagahora[2], Munetaka Ishiyama[1], Kosei Shioji[2], Kazumi Sasamoto[1], Kentaro Okuma[2], Shigeomi Shimizu[3] and Yuichiro Ueno[1]

[1]Dojindo Laboratories, Tabaru 2025-5, Mashiki-machi, Kumamoto 861-2202, Japan

[2]Department of Chemistry, Faculty of Science, Fukuoka University, Jonan-Ku, Fukuoka 814-0180, Japan

[3]Department of Pathological Cell Biology, Medical Research Institute, Tokyo Medical and Dental University, 1-5-45 Yushima, Bunkyo-ku, Tokyo 113-8510, Japan

Contents

Supplementary data	3
Syntheses of DALGreen and DAPGreen	12

Supplementary data

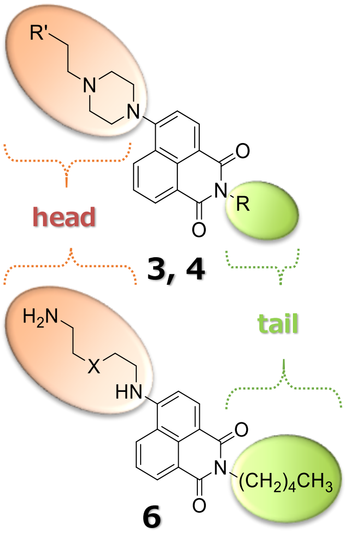

Compd.	R	R’	X
**3g**	$(CH_2)_4CH_3$	CH_3	-
**4a**	$(CH_2)_2CH_3$	NH_3Cl	-
**DALGreen (4b)**	$(CH_2)_4CH_3$	NH_3Cl	-
**4c**	$(CH_2)_6CH_3$	NH_3Cl	-
**4d**	$(CH_2)_9CH_3$	NH_3Cl	-
**4e**	$(CH_2)_2NH_2$	NH_3Cl	-
**4f**	$(CH_2)_4NH_2$	NH_3Cl	-
**DAPGreen (6a)**	$(CH_2)_4CH_3$	-	CH_2
**6b**	$(CH_2)_4CH_3$	-	NMe

Fig. S1 Chemical structures of DALGreen, DAPGreen and their analogous compounds.

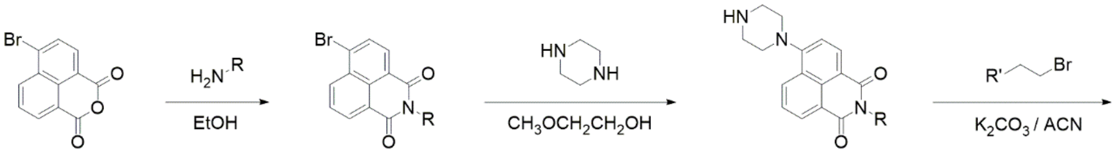

1a-f

1a (R= $(CH_2)_2CH_3$)
1b (R= $(CH_2)_4CH_3$)
1c (R= $(CH_2)_6CH_3$)
1d (R= $(CH_2)_9CH_3$)
1e (R= $(CH_2)_2$NHBoc)
1f (R= $(CH_2)_4$NHBoc)

2a-f

2a (R= $(CH_2)_2CH_3$)
2b (R= $(CH_2)_4CH_3$)
2c (R= $(CH_2)_6CH_3$)
2d (R= $(CH_2)_9CH_3$)
2e (R= $(CH_2)_2$NHBoc)
2f (R= $(CH_2)_4$NHBoc)

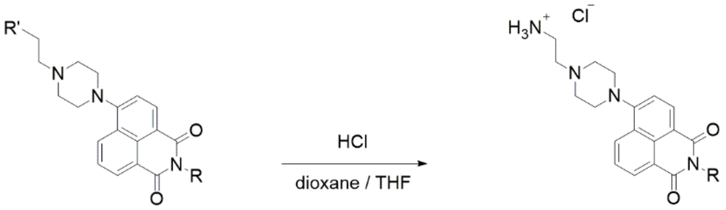

3a-g

3a (R= $(CH_2)_2CH_3$, R'=NHBoc)
3b (R= $(CH_2)_4CH_3$, R'=NHBoc)
3c (R= $(CH_2)_6CH_3$, R'=NHBoc)
3d (R= $(CH_2)_9CH_3$, R'=NHBoc)
3e (R= $(CH_2)_2$NHBoc, R'=NHBoc)
3f (R= $(CH_2)_4$NHBoc, R'=NHBoc)
3g (R= $(CH_2)_4CH_3$, R'=CH_3)

4a-f

4a (R= $(CH_2)_2CH_3$)
4b (R= $(CH_2)_4CH_3$)
4c (R= $(CH_2)_6CH_3$)
4d (R= $(CH_2)_9CH_3$)
4e (R= $(CH_2)_2NH_2$)
4f (R= $(CH_2)_4NH_2$)

Scheme S1. Synthesis of DALGreen (**4b**) and its analogous compounds.

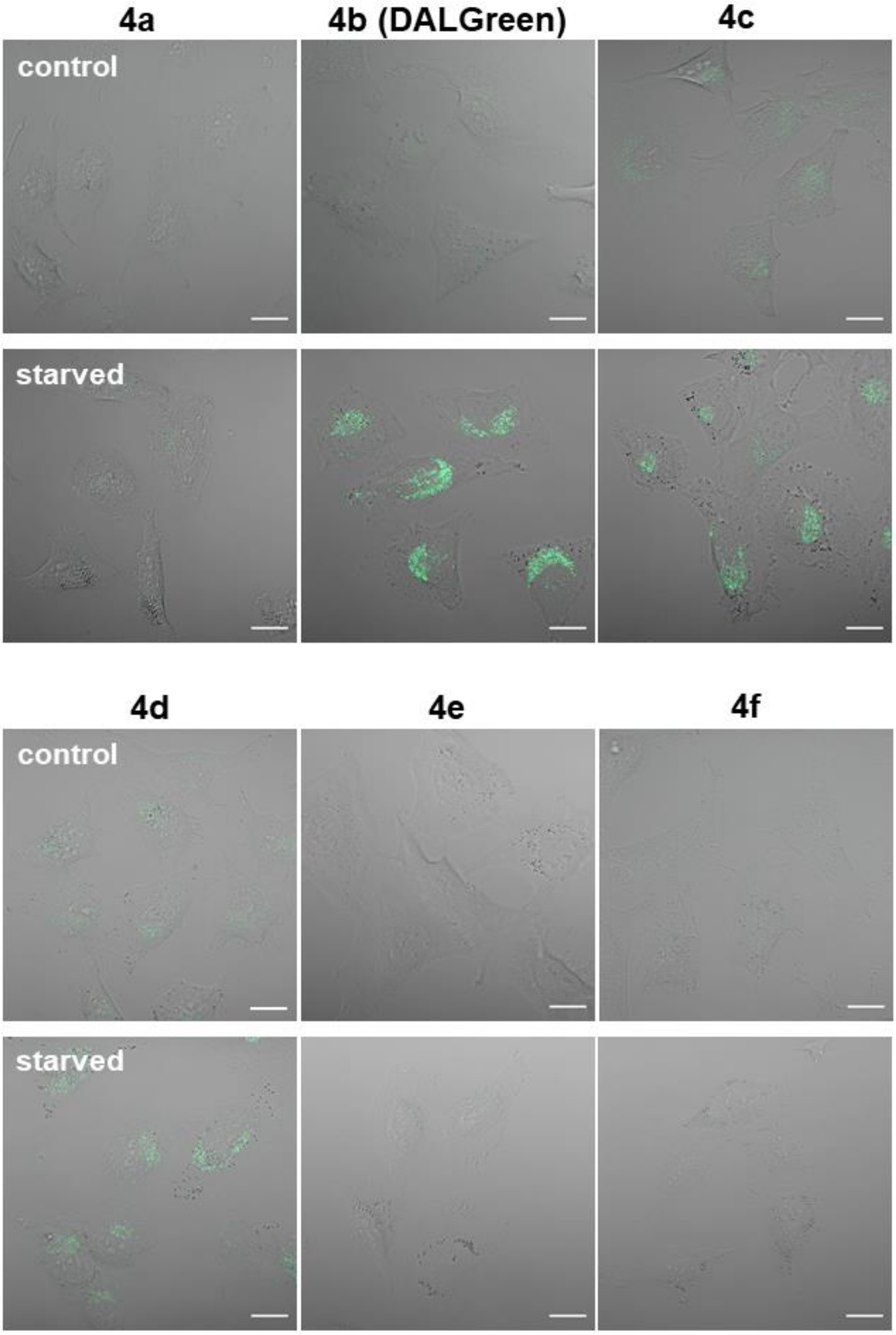

Fig. S2 Fluorescence images of DALGreen and its analogs (1 μM) with HeLa cells under nutrient-rich (control) or nutrient-deprived condition (starved, 5 h). Scale bar = 10 μm.

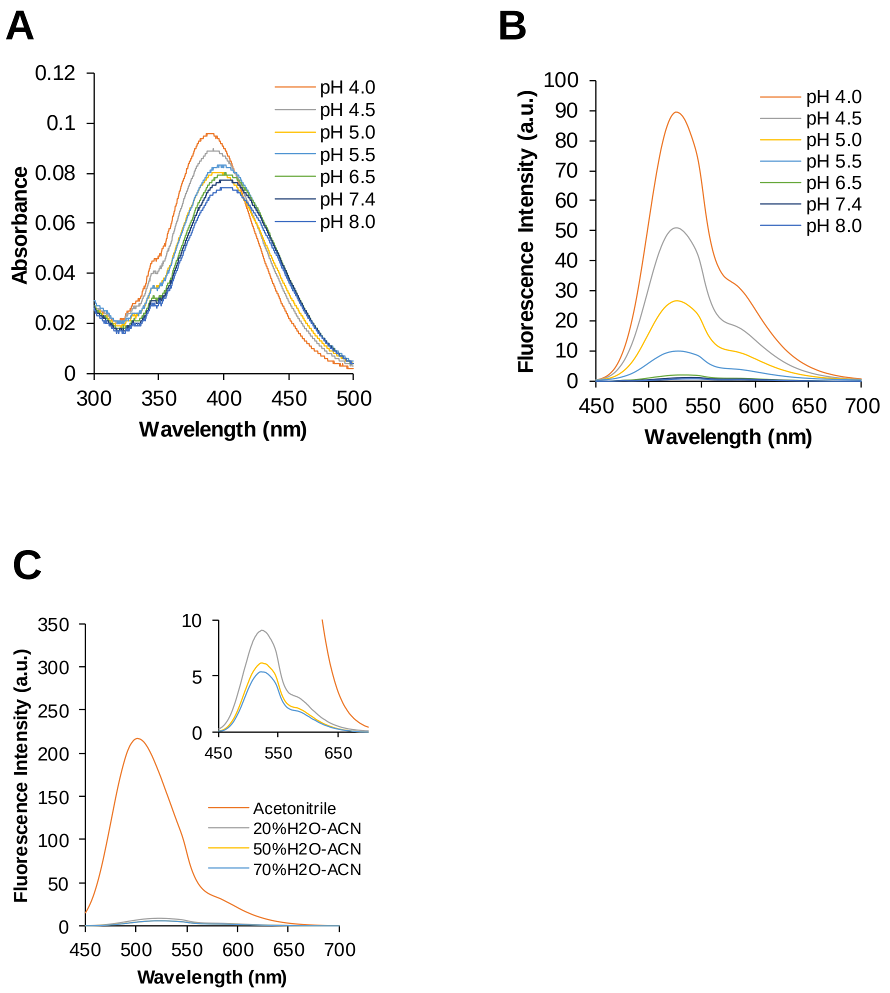

Fig. S3 Absorption (A) and emission (B) spectra of DALGreen (5.0 μM) in buffer solutions (pH 4.0 – 8.0), excited at 405 nm. (C) Fluorescence spectra of DALGreen (5.0 μM) excited at 405 nm in aqueous acetonitrile (ACN) solutions. A working solution of DALGreen (1.0 mM in DMSO) was diluted with MES buffer.

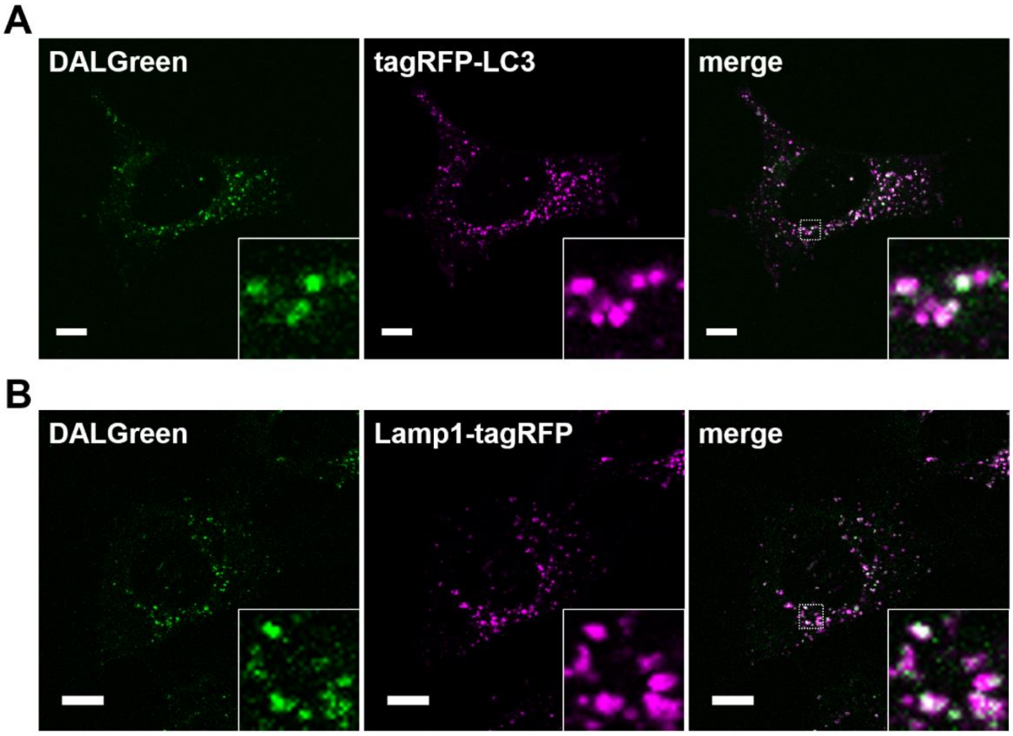

Fig. S4 Confocal microscopic images of DALGreen (1.0 µM). (A) Co-staining with tagRFP-LC3 as an autophagosomal marker expressed in MEF cells. (B) Co-staining with Lamp1-tagRFP as a lysosomal marker expressed in MEF cells.

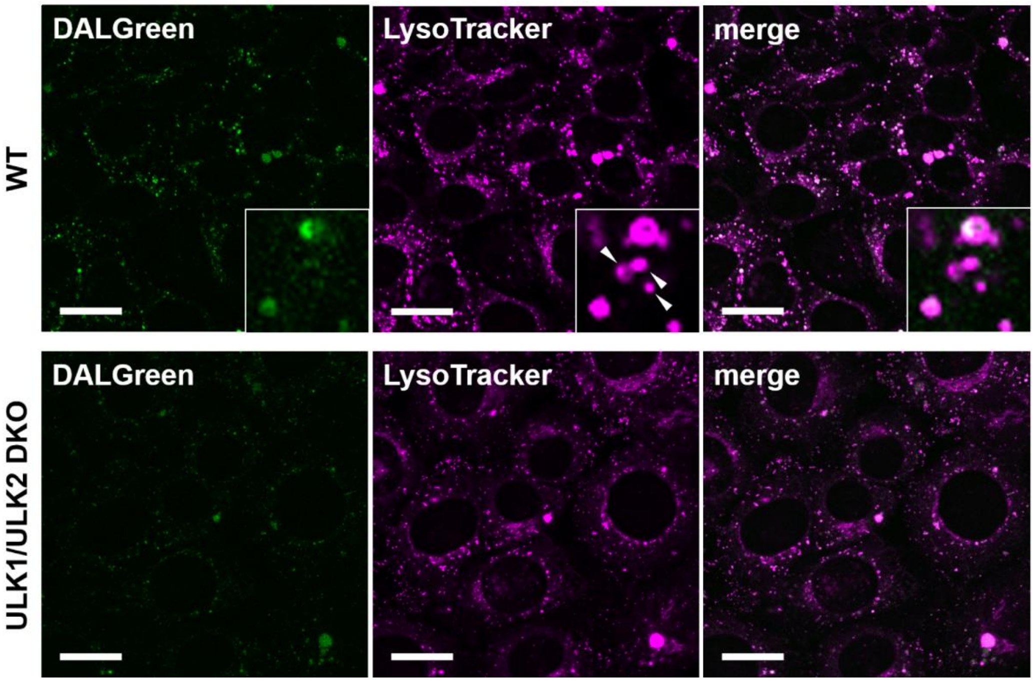

Fig. S5 Confocal microscopic images of wild-type (upper panel) and ULK1/2 double-knockout MEF cells (bottom panel), co-stained with DALGreen (1.0 µM) and LysoTracker (0.1 µM).

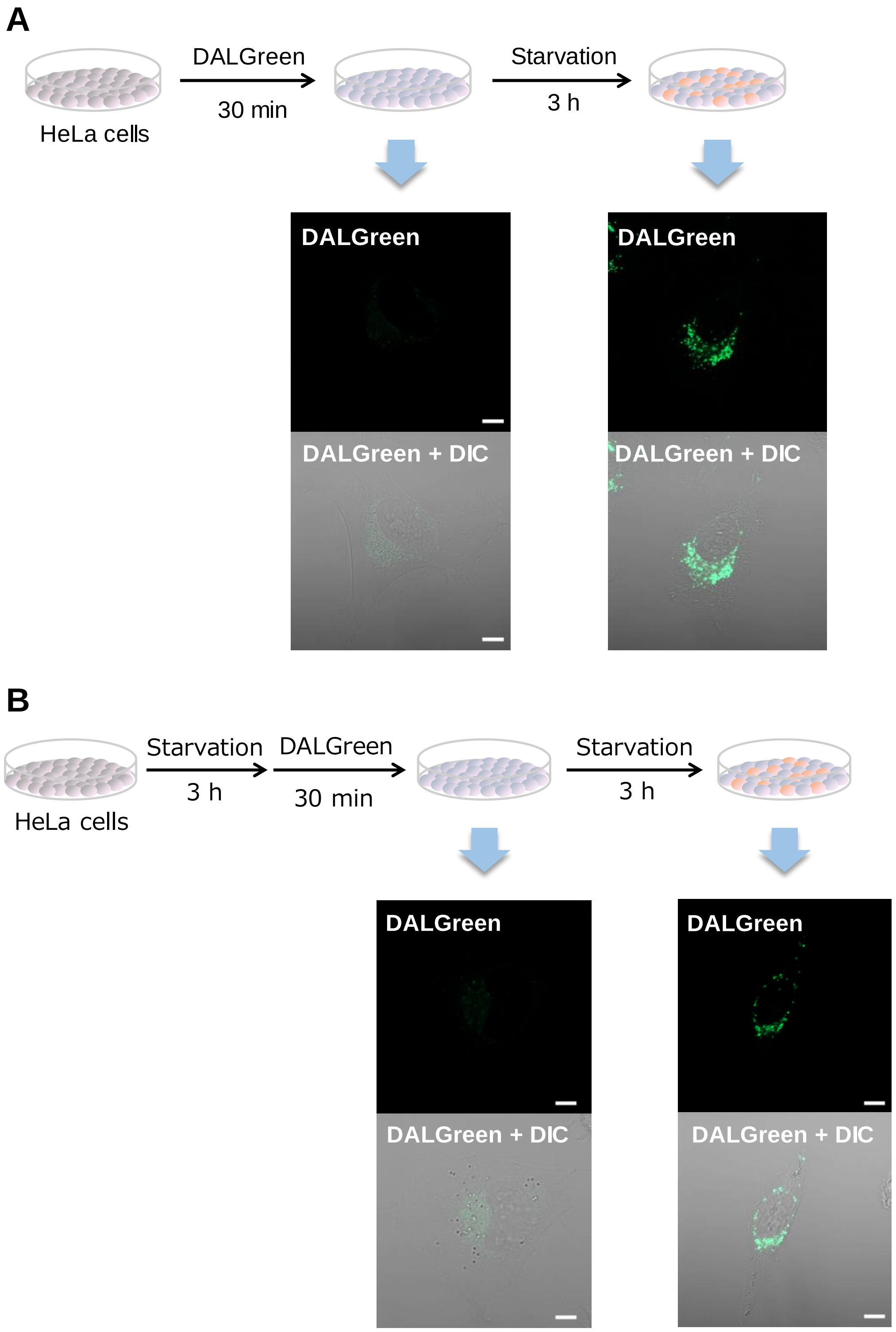

Fig. S6 Reversed staining procedure with DALGreen. (A) Normal procedure in which DALGreen was added before autophagy induction. (B) Reversed procedure in which DALGreen was added to autophagy-induced cells. Scale bar = 10 μm.

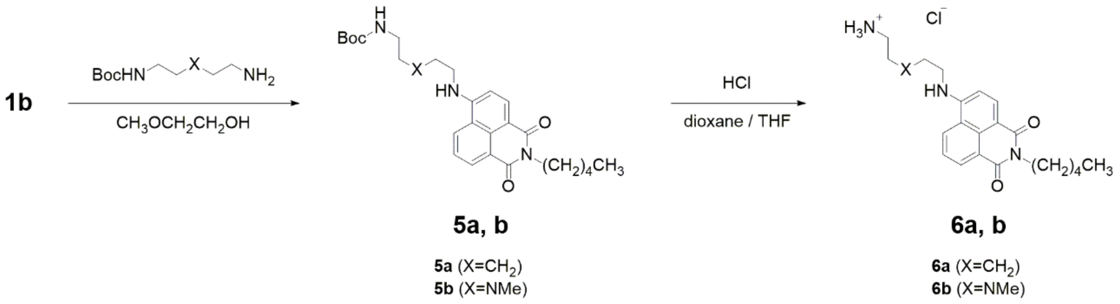

Scheme S2. Synthesis of DAPGreen (**6a**) and **6b**.

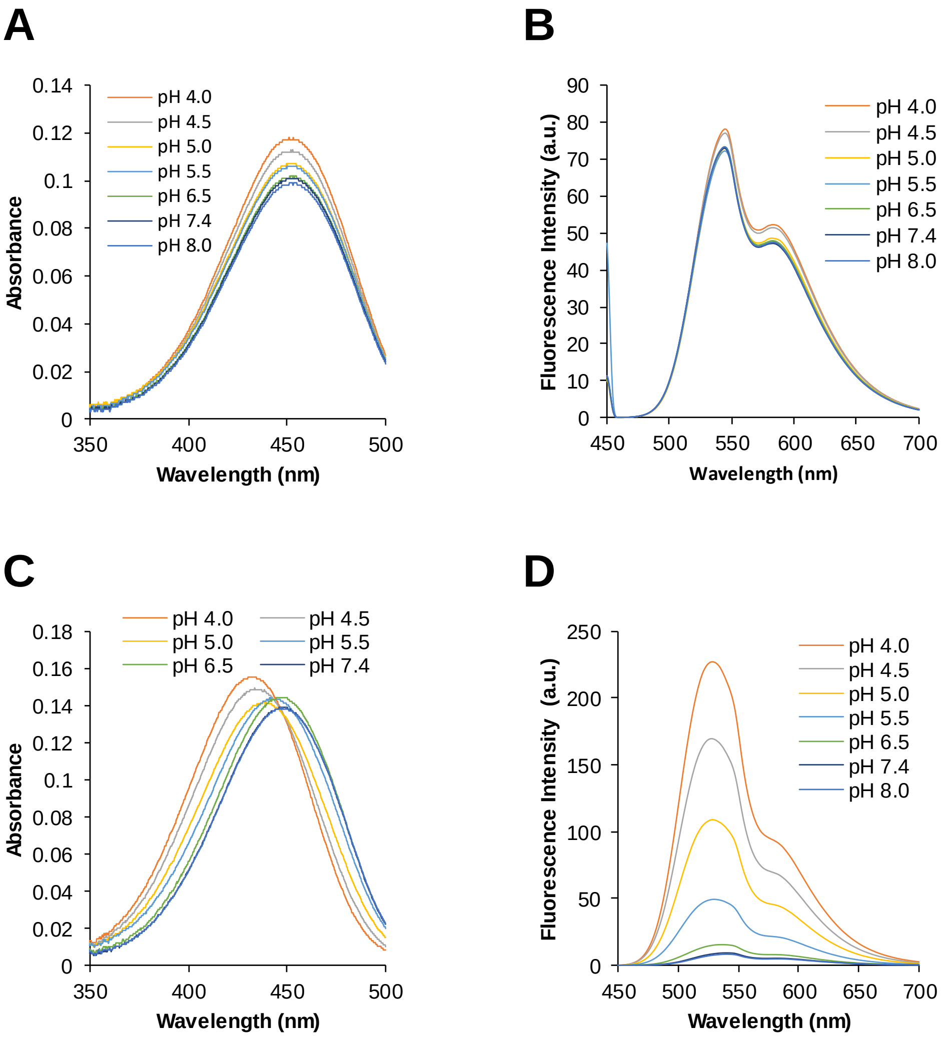

Fig. S7 Absorption and emission spectra of DAPGreen (A and B, respectively) and **6b** (C and D, respectively), excited at 450 nm in buffer solutions (pH 4.0 – 8.0). A working solution of DAPGreen (1.0 mM in DMSO) was diluted with MES buffer at the concentration of 5.0 μM.

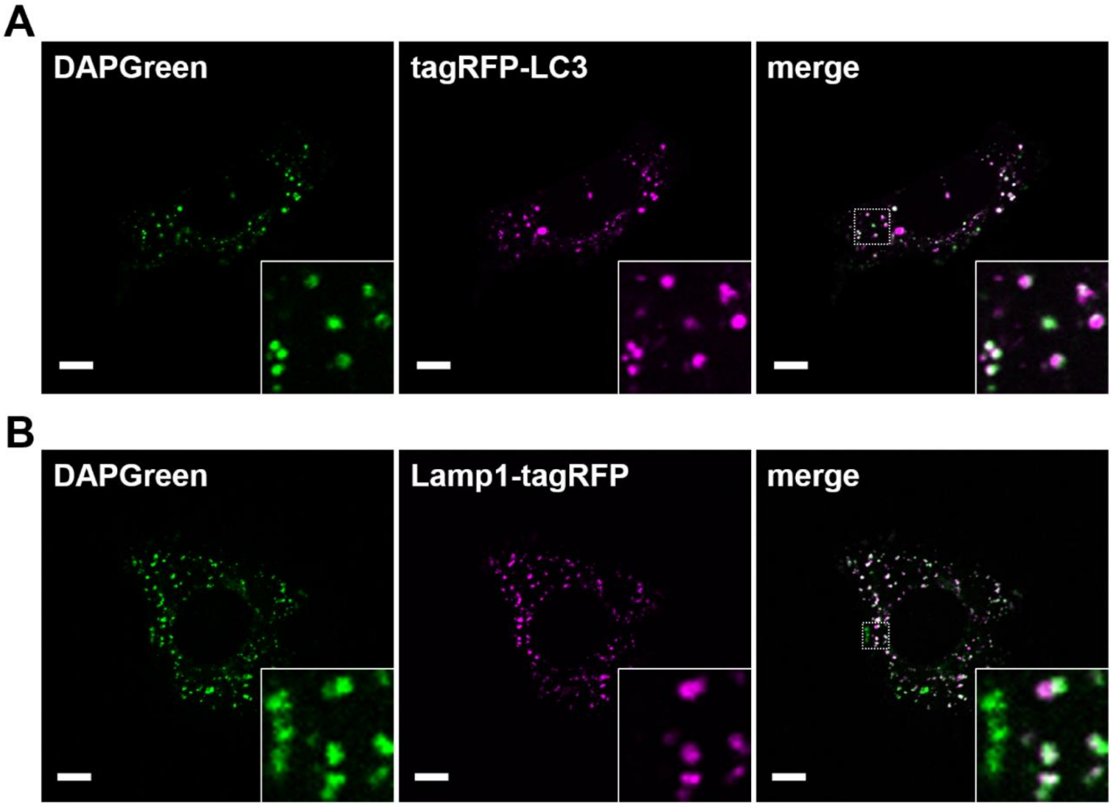

Fig. S8 Confocal microscopic images of DAPGreen (0.1 μM). (A) Co-staining with tagRFP-LC3 as an autophagosomal marker expressed in MEF cells. (B) Co-staining with Lamp1-tagRFP as a lysosomal marker expressed in MEF cells.

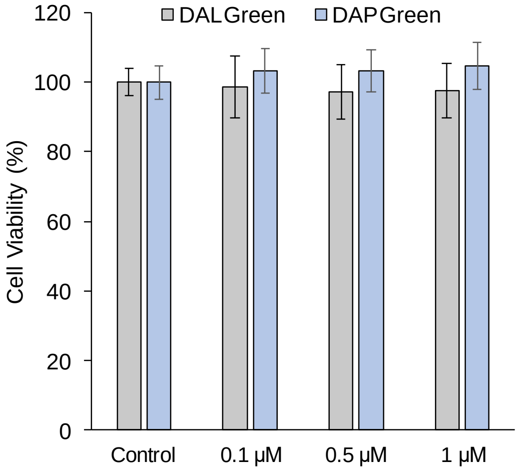

Fig. S9 Cell viabilities of DALGreen and DAPGreen for HeLa cells measured by CCK-8. The cell viability is shown as the percentage of the control.

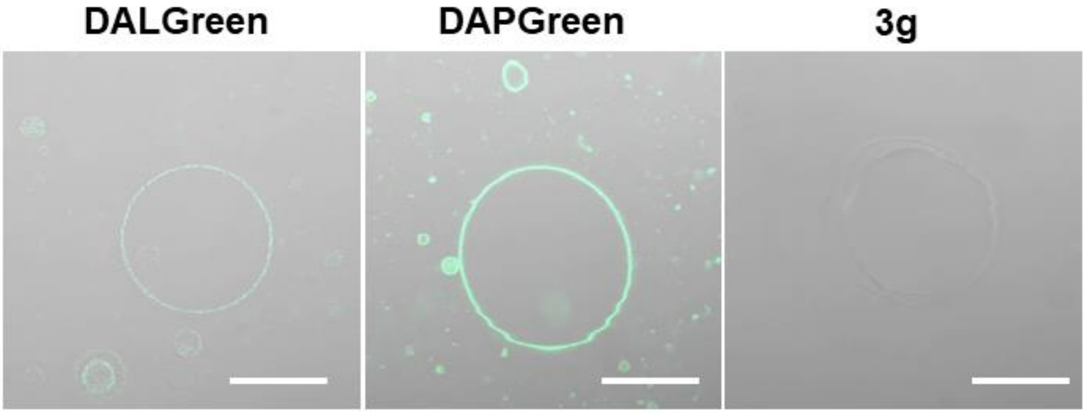

Fig. S10 Confocal microscopic images of liposomes treated with DALGreen, DAPGreen or **3g** in the formation of double membrane. Scale bar: 20 μm.

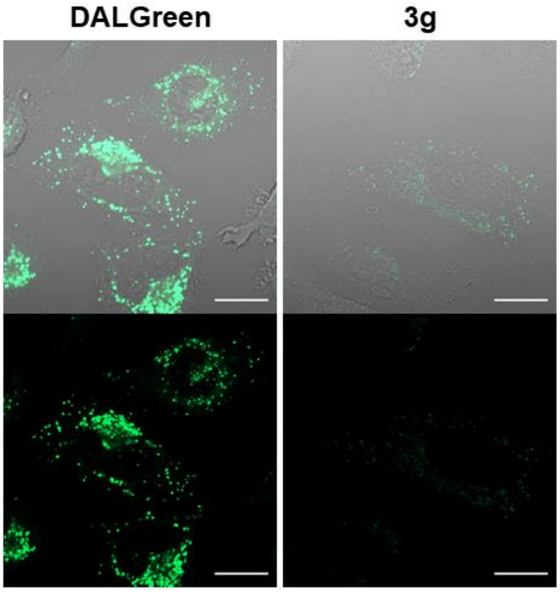

Fig. S11 Live-cell imaging of starved HeLa cells stained with DALGreen or **3g** for 5h. The upper panel shows the merged images of transfer and fluorescence, and the lower panel shows fluorescence only. Scale bar: 20 μm.

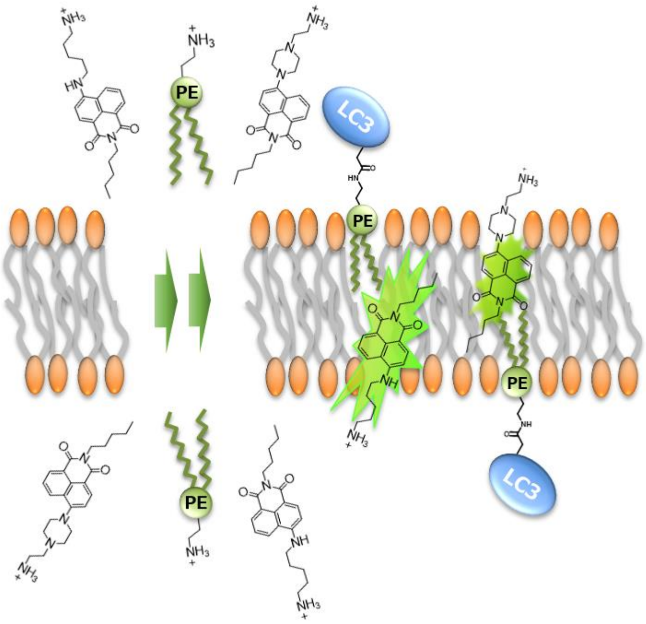

Fig. S12 A proposed staining mechanism of autophagosomal membrane with DALGreen or DAPGreen

Syntheses of DALGreen and DAPGreen

6-bromo-2-propyl-1*H*-benzo[*de*]isoquinoline-1,3(2*H*)-dione (1a)

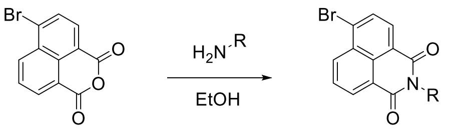

1a-f

A mixture of 4-bromo-1,8-naphthalic anhydride (1.0 g, 3.6 mmol), propylamine (298 mg, 5.04 mmol, 1.4 eq) and DMAP (528 mg, 4.3 mmol, 1.2 eq) in EtOH (50 mL) was stirred at 80 °C for 16 h. After the reaction mixture was cooled, the precipitate was filtered and dried to yield 930 mg (84%) of **1a** as a yellow solid. ^{1}H-NMR (400 MHz, $CDCl_3$) δ: 8.65 (d, 1H, *J* = 7.2 Hz), 8.55 (d, 1H, *J* = 8.5 Hz), 8.40 (d, 1H, *J* = 7.8 Hz), 8.03 (d, 1H, *J* = 7.8 Hz), 7.84 (t, 1H, *J* = 7.8 Hz), 4.13 (t, 2H, *J* = 7.5 Hz), 1.79-1.71 (m, 2H), 1.01 (t, 3H, *J* = 7.3 Hz); ^{13}C-NMR (101 MHz, $CDCl_3$): δ 163.5, 133.1, 131.9, 131.1, 131.0, 130.5, 130.1, 128.9, 128.0, 123.1, 122.2, 42.0, 21.3, 11.5.

6-bromo-2-pentyl-1*H*-benzo[*de*]isoquinoline-1,3(2*H*)-dione (1b)

Compound **1b** was prepared in the same manner as for **1a** with amylamine, instead of propylamine, in 80% yield as a yellow solid. ^{1}H-NMR (400 MHz, $CDCl_3$) δ: 8.65 (d, 1H, *J* = 7.2 Hz), 8.56 (d, 1H, *J* = 8.5 Hz), 8.40 (d, 1H, *J* = 7.8 Hz), 8.03 (d, 1H, *J* = 7.8 Hz), 7.84 (t, 1H, *J* = 7.8 Hz), 4.16 (t, 2H, *J* = 7.5 Hz), 1.75-1.69 (m, 2H), 1.42-1.38 (m, 4H), 0.91 (t, 3H, *J* = 7.3 Hz); ^{13}C-NMR (101 MHz, $CDCl_3$): δ 163.5, 133.1, 131.9, 131.1, 131.0, 130.5, 130.1, 128.9, 128.0, 123.1, 122.3, 40.6, 29.2, 27.7 22.4, 14.0.

6-bromo-2-heptyl-1*H*-benzo[*de*]isoquinoline-1,3(2*H*)-dione (1c)

Compound **1c** was prepared in the same manner as for **1a** with heptylamine in 56%

yield as a yellow solid. ^{1}H-NMR (400 MHz, $CDCl_3$) δ: 8.65 (d, 1H, *J* = 7.2 Hz), 8.56 (d, 1H, *J* = 8.5 Hz), 8.41 (d, 1H, *J* = 7.8 Hz), 8.03 (d, 1H, *J* = 7.8 Hz), 7.84 (t, 1H, *J* = 7.8 Hz), 4.16 (t, 2H, *J* = 7.5 Hz), 1.76-1.68 (m, 2H), 1.43-1.30 (m, 8H), 0.89 (t, 3H, *J* = 7.3 Hz); ^{13}C-NMR (101 MHz, $CDCl_3$): δ 163.5, 133.1, 131.9, 131.1, 131.0, 130.5, 130.1, 128.9, 128.0, 123.1, 122.2, 40.6, 31.7, 29.0, 28.0, 27.0 22.6, 14.0.

6-bromo-2-decyl-1*H*-benzo[*de*]isoquinoline-1,3(2*H*)-dione (1d)

Compound **1d** was prepared in the same manner as for **1a** with 1-aminodecane in 50% yield as a yellow solid. ^{1}H-NMR (400 MHz, $CDCl_3$) δ: 8.65 (d, 1H, *J* = 7.2 Hz), 8.56 (d, 1H, *J* = 8.5 Hz), 8.41 (d, 1H, *J* = 7.8 Hz), 8.04 (d, 1H, *J* = 7.8 Hz), 7.84 (t, 1H, *J* = 7.8 Hz), 4.16 (t, 2H, *J* = 7.5 Hz), 1.76-1.68 (m, 2H), 1.45-1.25 (m, 17H), 0.88 (t, 3H, *J* = 7.3 Hz); ^{13}C-NMR (101 MHz, $CDCl_3$): δ 163.5, 133.1, 131.9, 131.1, 131.0, 130.5, 130.1, 128.9, 128.0, 123.1, 122.2, 40.6, 31.9, 29.5, 29.3, 28.0, 27.1 22.6, 14.1.

***tert*-butyl-(2-(6-bromo-1,3-dioxo-1*H*-benzo[*de*]isoquinolin-2(3*H*)-yl)ethyl)-carbamate (1e)**

Compound **1e** was prepared in the same manner as for **1a** with *N*-(*tert*-butoxycarbonyl)-1,2-diaminoethane in 86% yield as a yellow solid. ^{1}H-NMR (400 MHz, $CDCl_3$) δ: 8.66 (d, 1H, *J* = 7.2 Hz), 8.57 (d, 1H, *J* = 8.5 Hz), 8.41 (d, 1H, *J* = 7.8 Hz), 8.04 (d, 1H, *J* = 7.8 Hz), 7.84 (t, 1H, *J* = 7.8 Hz), 4.93 (s, 1H), 4.35 (t, 2H, *J* = 7.5 Hz), 3.54-3.53 (m, 2H), 1.27 (s, 9H, *J* = 7.3 Hz); ^{13}C-NMR (101 MHz, $CDCl_3$): δ 163.9, 156.0, 133.3, 132.2, 131.3, 131.0, 130.5, 130.3, 129.0, 128.0, 122.8, 122.0, 79.1, 40.0, 39.5, 28.2.

tert-butyl-(4-(6-bromo-1,3-dioxo-1*H*-benzo[*de*]isoquinolin-2(3*H*)-yl)butyl)-carbamate (1f)

Compound **1f** was prepared in the same manner as for **1a** with *N*-(*tert*-butoxycarbonyl)-1,4-diaminobutane in 56% yield as a yellow solid. ^{1}H-NMR (400 MHz, $CDCl_3$) δ: 8.65 (d, 1H, J = 7.2 Hz), 8.57 (d, 1H, J = 8.5 Hz), 8.41 (d, 1H, J = 7.8 Hz), 8.04 (d, 1H, J = 7.8 Hz), 7.85 (t, 1H, J = 7.8 Hz), 4.62 (s, 1H), 4.18 (t, 2H, J = 7.5 Hz), 3.20-3.18 (m, 2H), 1.79-1.75 (m, 2H), 1.62-1.57 (m, 2H), 1.42 (s, 9H, J = 7.3 Hz); ^{13}C-NMR (101 MHz, $CDCl_3$): δ 163.5, 155.9, 133.2, 132.0, 131.2, 131.0, 130.5, 130.2, 128.9, 128.0, 123.0, 122.1, 79.0, 40.2, 40.0, 28.4, 27.5, 25.4.

6-(piperazin-1-yl)-2-propyl-1*H*-benzo[de]isoquinoline-1,3(2*H*)-dione (2a)

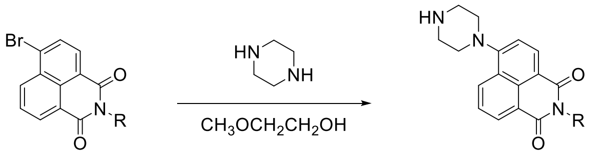

1a-f **2a-f**

To a stirred solution of **1a** (500 mg, 1.5 mmol) in 2-methoxyethanol (50 mL) was added piperazine (1.3 g, 15 mmol, 10 eq). The resulting mixture was stirred at 120 °C for 16 h and was evaporated *in vacuo*. The crude product was purified by silica-gel column chromatography with $CHCl_3$ / MeOH (7/3, v/v) eluent to yield 400 mg (82%) of **2a** as a yellow solid. ^{1}H-NMR (400 MHz, $CDCl_3$) δ: 8.58 (d, 1H, J = 7.2 Hz), 8.52 (d, 1H, J = 8.5 Hz), 8.42 (d, 1H, J = 7.8 Hz), 7.69 (t, 1H, J = 7.8 Hz), 7.21 (d, 1H, J = 7.8 Hz), 4.13 (t, 2H, J = 7.5 Hz), 3.22 (d, 8H, J = 7.8 Hz), 1.78-1.73 (m, 2H), 1.01 (t, 3H, J = 7.3 Hz); ^{13}C-NMR (101 MHz, $CDCl_3$): δ 164.5, 164.0, 156.3, 132.5, 131.0, 130.2, 129.9, 126.2, 125.6, 123.3, 116.7, 114.9, 54.4, 46.2, 41.7, 21.4, 11.5; ESI-MS: calcd for $[M]^+$, 323.1;

found, 323.6.

2-pentyl-6-(piperazin-1-yl)-1*H*-benzo[de]isoquinoline-1,3(2*H*)-dione (2b)

Compound **2b** was prepared in the same manner as for **2a** from **1b** in 79% yield as a yellow solid. ^{1}H-NMR (400 MHz, $CDCl_3$) δ: 8.56 (d, 1H, *J* = 8.5 Hz), 8.51 (d, 1H, *J* = 7.8 Hz), 8.41 (d, 1H, *J* = 7.8 Hz), 7.69 (t, 1H, *J* = 7.8 Hz), 7.21 (d, 1H, *J* = 7.2 Hz), 4.15 (t, 2H, *J* = 7.5 Hz), 3.24-3.21 (m, 2H), 1.91 (s, 2H), 1.72 (m, 2H), 1.39 (s, 4H), 0.91 (t, 3H, *J* = 7.3 Hz); ^{13}C-NMR (101 MHz, $CDCl_3$): δ 164.4, 163.9, 156.3, 132.5, 131.0 130.2, 129.8, 126.1, 125.5, 123.2, 116.7, 114.8, 54.4, 46.2, 40.2, 29.2, 27.8, 22.4, 14.0; ESI-MS: calcd for $[M]^+$, 351.1; found, 351.7.

2-heptyl-6-(piperazin-1-yl)-1*H*-benzo[de]isoquinoline-1,3(2*H*)-dione (2c)

Compound **2c** was prepared in the same manner as for **2a** from **1c** in 79% yield as a yellow solid. ^{1}H-NMR (400 MHz, $CDCl_3$) δ: 8.57 (d, 1H, *J* = 8.5 Hz), 8.51 (d, 1H, *J* = 7.8 Hz), 8.41 (d, 1H, *J* = 7.8 Hz), 7.69 (t, 1H, *J* = 7.8 Hz), 7.21 (d, 1H, *J* = 7.2 Hz), 4.15 (t, 2H, *J* = 7.5 Hz), 3.25-3.20 (m, 8H), 1.75-1.68 (m, 2H), 1.43-1.28 (m, 8H), 0.89 (t, 3H, *J* = 7.3 Hz); ^{13}C-NMR (101 MHz, $CDCl_3$): δ 164.3, 163.8, 156.2, 132.4, 130.9 130.1, 129.7, 126.3, 126.0, 125.5, 123.2, 116.6, 114.8, 54.3, 46.2, 40.2, 31.7, 31.5, 29.0, 28.8, 28.1, 27.2, 27.1, 22.7, 22.5, 22.4, 14.0; ESI-MS: calcd for $[M]^+$, 379.2; found, 379.7.

2-decyl-6-(piperazin-1-yl)-1*H*-benzo[de]isoquinoline-1,3(2*H*)-dione (2d)

Compound **2d** was prepared in the same manner as for **2a** from **1d** in 79% yield as a yellow solid. ^{1}H-NMR (400 MHz, $CDCl_3$) δ: 8.57 (d, 1H, *J* = 8.5 Hz), 8.51 (d, 1H, *J* = 7.8 Hz), 8.41 (d, 1H, *J* = 7.8 Hz), 7.69 (t, 1H, *J* = 7.8 Hz), 7.22 (d, 1H, *J* = 7.2 Hz), 4.15

(t, 2H, *J* = 7.5 Hz), 3.25-3.20 (m, 8H), 1.75-1.67 (m, 2H), 1.44-1.25 (m, 14H), 0.88 (t, 3H, *J* = 7.3 Hz); ^{13}C-NMR (101 MHz, $CDCl_3$): δ 164.3, 163.9, 156.2, 132.4, 130.9 130.1, 129.8, 126.1, 125.8, 125.5, 123.2, 116.7, 114.8, 54.3, 46.2, 40.3, 31.8, 31.6, 29.5, 29.3, 29.2, 28.1, 27.1, 22.6, 22.4, 14.1; ESI-MS: calcd for $[M+H]^+$, 422.2; found, 422.2.

***tert*-butyl(2-(1,3-dioxo-6-(piperazin-1-yl)-1*H*-benzo[de]isoquinolin-2(3*H*)-yl)ethyl)-carbamate (2e)**

Compound **2e** was prepared in the same manner as for **2a** from **1e** in 82% yield as a yellow solid. ^{1}H-NMR (400 MHz, $CDCl_3$) δ: 8.57 (d, 1H, *J* = 8.5 Hz), 8.51 (d, 1H, *J* = 7.8 Hz), 8.40 (d, 1H, *J* = 7.8 Hz), 7.68 (t, 1H, *J* = 7.8 Hz), 7.20 (d, 1H, *J* = 7.2 Hz), 5.08 (s, 1H), 4.34 (t, 2H, *J* = 7.5 Hz), 3.52-3.51 (m, 2H), 3.23-3.21 (m, 8H), 1.30 (s, 9H); ^{13}C-NMR (101 MHz, $CDCl_3$): δ 164.8, 164.3, 156.5, 156.0, 132.8, 131.3, 130.4, 129.9, 126.1, 125.6, 123.0, 116.3, 114.9, 79.0, 54.3, 46.2, 39.9, 39.6, 28.2; ESI-MS: calcd for $[M+Na]^+$, 447.2; found, 447.1.

***tert*-butyl(4-(6-(1l3,4-bromazinan-1-yl)-1,3-dioxo-1*H*-benzo[de]isoquinolin-2(3*H*)-yl)butyl)carbamate (2f)**

Compound **2f** was prepared in the same manner as for **2a** from **1f** in 66% yield as a yellow solid. ^{1}H-NMR (400 MHz, $CDCl_3$) δ: 8.57 (d, 1H, *J* = 8.5 Hz), 8.50 (d, 1H, *J* = 7.8 Hz), 8.41 (d, 1H, *J* = 7.8 Hz), 7.69 (t, 1H, *J* = 7.8 Hz), 7.21 (d, 1H, *J* = 7.2 Hz), 4.67 (s, 1H), 4.18 (t, 2H, *J* = 7.5 Hz), 3.25-3.18 (m, 10H), 1.78-1.74 (m, 2H), 1.62-1.58 (m, 2H), 1.42 (s, 9H); ^{13}C-NMR (101 MHz, $CDCl_3$): δ 164.5, 164.0, 156.4, 155.9, 132.6, 131.1, 130.3, 129.9, 126.1, 125.6, 123.2, 116.6, 114.9, 79.0, 54.4, 46.2, 40.2, 39.7, 28.4, 27.5, 25.4; ESI-MS: calcd for $[M]^+$, 453.2; found, 453.4.

tert-butyl(2-(4-(1,3-dioxo-2-propyl-2,3-dihydro-1_H_-benzo[de]isoquinolin-6-yl)-piperazin-1-yl)ethyl)carbamate (3a)

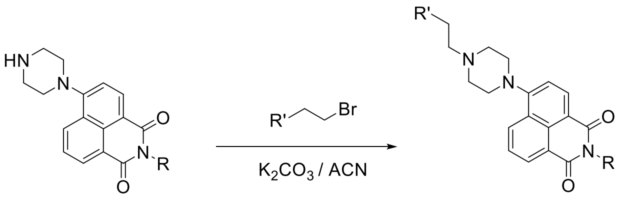

2a-f **3a-g**

A mixture of **2a** (500 mg, 1.5 mmol), 2-(Boc-amino)ethyl bromide (827 mg, 3.7 mmol, 2.5 eq) and potassium carbonate (510 mg, 3.7 mmol, 2.5 eq) in acetonitrile (50 mL) was stirred at 90 °C for 16 h. After being cooled, the reaction mixture was filtered and the filtrate was evaporated. The crude product was purified by silica-gel column chromatography with $CHCl_3$ / MeOH (9/1, v/v) eluent to yield 380 mg (54%) of **3a** as a yellow solid. ^{1}H-NMR (400 MHz, $CDCl_3$) δ: 8.58 (d, 1H, J = 7.2 Hz), 8.51 (d, 1H, J = 8.5 Hz), 8.39 (d, 1H, J = 7.8 Hz), 7.68 (t, 1H, J = 7.8 Hz), 7.21 (d, 1H, J = 7.8 Hz), 5.01 (s, 1H), 4.13 (t, 2H, J = 7.5 Hz), 3.29 (m, 6H), 2.79 (s, 4H), 2.62 (t, 2H, J = 7.3 Hz), 1.78-1.73 (m, 2H), 1.47 (s, 9H), 1.00 (t, 3H, J = 7.3 Hz); ^{13}C-NMR (101 MHz, $CDCl_3$): δ 164.4, 163.9, 155.9, 155.8, 132.4, 131.0, 130.1, 129.8, 126.1, 125.6, 123.2, 116.7, 114.8, 79.2, 61.9, 57.2, 52.9, 42.3, 41.7, 37.1, 28.4, 23.2, 21.4, 11.5; ESI-MS: calcd for $[M]^+$, 466.2; found, 466.6.

tert-butyl(2-(4-(1,3-dioxo-2-pentyl-2,3-dihydro-1_H_-benzo[de]isoquinolin-6-yl)-piperazin-1-yl)ethyl)carbamate (3b)

Compound **3b** was prepared in the same manner as for **3a** from **2b** in 48% yield as a yellow solid. ^{1}H-NMR (400 MHz, $CDCl_3$) δ: 8.58 (d, 1H, J = 8.5 Hz), 8.51 (d, 1H, J =

7.8 Hz), 8.39 (d, 1H, *J* = 7.8 Hz), 7.68 (t, 1H, *J* = 7.8 Hz), 7.21 (d, 1H, *J* = 7.2 Hz), 4.99 (s, 1H), 4.15 (t, 2H, *J* = 7.5 Hz), 3.29 (s, 6H), 2.78 (s, 4H), 2.62 (m, 2H), 1.72 (m, 2H), 1.47 (s, 9H), 1.39 (s, 2H), 1.39 (s, 4H), 0.91 (t, 3H, *J* = 7.3 Hz); ^{13}C-NMR (101 MHz, $CDCl_3$): δ 164.4, 164.0, 155.9, 155.8, 132.5, 131.0 130.1, 129.8, 126.1, 125.6, 123.3, 116.8, 114.9, 79.3, 57.2, 53.0, 45.7, 40.3, 37.1, 29.7, 29.2, 28.4, 27.8, 22.4, 14.0; ESI-MS: calcd for $[M]^+$, 494.2; found, 494.7.

***tert*-butyl(2-(4-(2-heptyl-1,3-dioxo-2,3-dihydro-1*H*-benzo[de]isoquinolin-6-yl)-piperazin-1-yl)ethyl)carbamate (3c)**

Compound **3c** was prepared in the same manner as for **3a** from **2c** in 53% yield as a yellow solid. ^{1}H-NMR (400 MHz, $CDCl_3$) δ: 8.56 (d, 1H, *J* = 8.5 Hz), 8.51 (d, 1H, *J* = 7.8 Hz), 8.39 (d, 1H, *J* = 7.8 Hz), 7.68 (t, 1H, *J* = 7.8 Hz), 7.21 (d, 1H, *J* = 7.2 Hz), 4.99 (s, 1H), 4.15 (t, 2H, *J* = 7.5 Hz), 3.29 (s, 6H), 2.78 (s, 4H), 2.62 (t, 2H, *J* = 7.8 Hz), 1.75-1.68 (m, 2H), 1.47 (s, 9H), 1.43-1.28 (m, 8H), 0.87 (t, 3H, *J* = 7.3 Hz); ^{13}C-NMR (101 MHz, $CDCl_3$): δ 164.4, 164.0, 155.9, 155.8, 132.4, 131.0 130.1, 129.8, 126.1, 125.6, 123.3, 116.8, 114.9, 79.3, 57.2, 53.0, 40.3, 31.7, 29.6, 29.0, 28.4, 28.1, 27.9, 27.1, 23.2, 22.6, 14.0; ESI-MS: calcd for $[M]^+$, 522.3; found, 522.8.

***tert*-butyl(2-(4-(2-decyl-1,3-dioxo-2,3-dihydro-1*H*-benzo[de]isoquinolin-6-yl)-piperazin-1-yl)ethyl)carbamate (3d)**

Compound **3d** was prepared in the same manner as for **3a** from **2d** in 50% yield as a yellow solid. ^{1}H-NMR (400 MHz, $CDCl_3$) δ: 8.58 (d, 1H, *J* = 8.5 Hz), 8.51 (d, 1H, *J* = 7.8 Hz), 8.39 (d, 1H, *J* = 7.8 Hz), 7.68 (t, 1H, *J* = 7.8 Hz), 7.21 (d, 1H, *J* = 7.2 Hz), 4.99 (s, 1H), 4.15 (t, 2H, *J* = 7.5 Hz), 3.29 (s, 6H), 2.78 (s, 4H), 2.62 (t, 2H, *J* = 7.8 Hz),

1.75-1.67 (m, 2H), 1.47 (s, 9H), 1.44-1.25 (m, 14H), 0.88 (t, 3H, *J* = 7.3 Hz); ^{13}C-NMR (101 MHz, $CDCl_3$): δ 164.4, 163.9, 155.9, 155.8, 132.4, 131.0 130.1, 129.8, 126.1, 125.6, 123.3, 116.8, 114.8, 79.2, 61.2, 57.2, 53.0, 40.3, 37.1, 31.8, 29.5, 29.4, 29.3, 28.7, 28.4, 28.1, 27.9, 27.1, 22.6, 14.1; ESI-MS: calcd for $[M]^+$, 564.3; found, 564.7.

***tert*-butyl(2-(6-(4-(2-((tert-butoxycarbonyl)amino)ethyl)piperazin-1-yl)-1,3-dioxo-1*H*-benzo[de]isoquinolin-2(3*H*)-yl)ethyl)carbamate (3e)**

Compound **3e** was prepared in the same manner as for **3a** from **2e** in 54% yield as a yellow solid. ^{1}H-NMR (400 MHz, $CDCl_3$) δ: 8.58 (d, 1H, *J* = 8.5 Hz), 8.52 (d, 1H, *J* = 7.8 Hz), 8.40 (d, 1H, *J* = 7.8 Hz), 7.68 (t, 1H, *J* = 7.8 Hz), 7.21 (d, 1H, *J* = 7.2 Hz), 5.01 (s, 2H), 4.33 (bs, 2H), 3.52-3.51 (m, 2H), 3.29 (s, 6H), 2.79 (s, 4H), 2.62 (bs, 2H), 1.47 (s, 9H), 1.30 (s, 9H); ^{13}C-NMR (101 MHz, $CDCl_3$): δ 164.8, 164.3, 156.0, 155.9, 132.8, 131.3, 130.4, 130.0, 126.1, 125.6, 123.0, 116.5, 114.9, 79.3, 79.0, 57.2, 53.0, 52.9, 39.9, 39.6, 37.1, 28.4, 28.2; ESI-MS: calcd for $[M]^+$, 567.3; found, 567.6.

***tert*-butyl(4-(6-(4-(2-((tert-butoxycarbonyl)amino)ethyl)-1l3,4-bromazinan-1-yl)-1,3-dioxo-1*H*-benzo[de]isoquinolin-2(3*H*)-yl)butyl)carbamate (3f)**

Compound **3f** was prepared in the same manner as for **3a** from **2f** in 81% yield as a yellow solid. ^{1}H-NMR (400 MHz, $CDCl_3$) δ: 8.57(d, 1H, *J* = 8.5 Hz), 8.51 (d, 1H, *J* = 7.8 Hz), 8.40 (d, 1H, *J* = 7.8 Hz), 7.68 (t, 1H, *J* = 7.8 Hz), 7.21 (d, 1H, *J* = 7.2 Hz), 4.98 (s, 1H), 4.62 (s, 1H), 4.18 (t, 2H, *J* = 7.8 Hz), 3.29 (s, 6H), 3.19-3.18 (m, 2H), 2.79 (s, 4H), 2.62 (t, 2H, *J* = 7.8 Hz), 1.78-1.74 (m, 2H), 1.62-1.58 (m, 2H), 1.47 (s, 9H), 1.42 (s, 9H); ^{13}C-NMR (101 MHz, $CDCl_3$): δ 164.5, 164.0, 155.9, 132.6, 131.1, 130.3, 129.8, 126.1, 125.6, 123.2, 116.7, 114.9, 79.3, 79.0, 57.2, 53.0, 40.2, 39.7, 37.1, 28.4, 27.5,

25.4; ESI-MS: calcd for $[M]^+$, 595.3; found, 595.7.

2-pentyl-6-(4-propylpiperazin-1-yl)-1*H*-benzo[de]isoquinoline-1,3(2*H*)-dione (3g)

Compound **3g** was prepared in the same manner as for **3a** from **2b** with 1-iodopropane in 74% yield as a yellow solid. ^{1}H-NMR (400 MHz, $CDCl_3$) δ: 8.57 (d, 1H, *J* = 8.5 Hz), 8.51 (d, 1H, *J* = 7.8 Hz), 8.40 (d, 1H, *J* = 7.8 Hz), 7.68 (t, 1H, *J* = 7.8 Hz), 7.21 (d, 1H, *J* = 7.2 Hz), 4.15 (t, 2H, *J* = 7.5 Hz), 3.30 (s, 4H), 2.77 (s, 4H), 2.46 (t, 2H, *J* = 7.8 Hz), 1.74-1.70 (m, 2H), 1.62-1.56 (m, 2H), 1.40-1.35 (m, 4H), 0.96 (t, 3H, *J* = 7.3 Hz), 0.90 (t, 3H, *J* = 7.3 Hz); ^{13}C-NMR (101 MHz, $CDCl_3$): δ 164.4, 164.0, 155.9, 132.5, 131.0 130.2, 129.8, 126.1, 125.5, 123.2, 116.6, 114.8, 60.6, 53.2, 53.0, 40.2, 29.2, 27.8, 22.4, 20.0, 14.0, 11.9; HRMS (ESI^+): calcd for $[M+H]^+$, 394.24945; Found, 394.25049.

2-(4-(1,3-dioxo-2-propyl-2,3-dihydro-1*H*-benzo[de]isoquinolin-6-yl)piperazin-1-yl)-ethan-1-aminium chloride (4a)

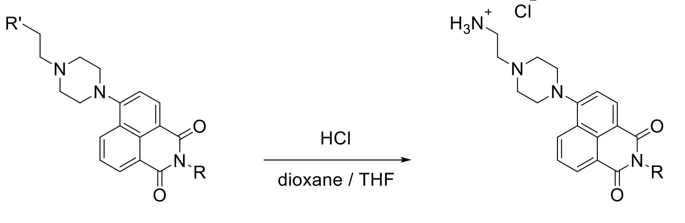

3a-f **4a-f**

To stirred solution of **3a** in THF (5 mL) was added 4N HCl in dioxane (5 mL), and the reaction mixture was stirred for 2 h at room temperature. The precipitate was filtered, washed with THF and then with $CHCl_3$ and dried over P_2O_5 to yield 100 mg (77%) of **4a** as a yellow solid. ^{1}H-NMR (400 MHz, CD_3OD) δ: 8.61-8.54 (m, 3H), 7.86 (t, 1H, *J* = 8.3 Hz), 7.50 (d, 1H, *J* = 7.3 Hz), 4.12 (t, 2H, *J* = 7.3 Hz), 3.71-3.56 (m, 12H), 1.80-1.71 (m, 2H), 1.01 (t, 3H, *J* = 7.3 Hz); ^{13}C-NMR (101 MHz, CD_3OD): δ 164.2,

163.7, 153.7, 131.9, 130.9, 129.8, 129.3, 126.3, 126.0, 122.9, 117.8, 115.8, 53.2, 52.5, 49.6, 41.3, 33.7, 20.9, 10.3; HRMS (ESI^+): calcd for $[M]^+$, 367.21340; Found, 367.21350.

2-(4-(1,3-dioxo-2-pentyl-2,3-dihydro-1*H*-benzo[de]isoquinolin-6-yl)piperazin-1-yl)-ethan-1-aminium chloride (4b)

Compound **4b** was prepared in the same manner as for **4a** from **3b** in 80% yield as a yellow solid. ^{1}H-NMR (400 MHz, CD_3OD) δ: 8.62-8.54 (m, 3H), 7.87 (t, 1H, J = 8.3 Hz), 7.50 (d, 1H, J = 7.3 Hz), 4.15 (t, 2H, J = 7.3 Hz), 3.68-3.50 (m, 12H), 1.73 (t, 2H, J = 8.3 Hz), 1.42 (s, 4H), 0.95 (t, 3H, J = 7.3 Hz); ^{13}C-NMR (101 MHz, CD_3OD): δ 164.1, 163.7, 153.7, 131.9, 130.9, 129.8, 129.3, 126.3, 126.0, 122.9, 117.8, 115.8, 53.2, 52.5, 49.6, 39.8, 33.7, 28.9, 27.3, 22.0, 12.9; HRMS (ESI^+): calcd for $[M]^+$, 395.24470; Found, 395.24596.

2-(4-(2-heptyl-1,3-dioxo-2,3-dihydro-1*H*-benzo[de]isoquinolin-6-yl)piperazin-1-yl)-ethan-1-aminium chloride (4c)

Compound **4c** was prepared in the same manner as for **4a** from **3c** in 70% yield as a yellow solid. ^{1}H-NMR (400 MHz, CD_3OD) δ: 8.50 (d, 1H, J = 8.3 Hz), 8.43 (d, 1H, J = 8.3 Hz), 7.80 (t, 1H, J = 8.3 Hz), 7.42 (d, 1H, J = 7.3 Hz), 4.08 (t, 2H, J = 7.3 Hz), 3.87-3.59 (m, 12H), 1.68 (t, 2H, J = 8.3 Hz), 1.41-1.32 (m, 8H), 0.91 (t, 3H, J = 7.3 Hz); ^{13}C-NMR (101 MHz, CD_3OD): δ 164.1, 163.7, 153.7, 131.8, 130.8, 129.8, 129.3, 126.3, 126.0, 122.8, 117.8, 115.7, 53.2, 52.5, 49.6, 39.8, 33.7, 31.5, 28.7, 27.6, 26.7, 22.2, 13.0; HRMS (ESI^+): calcd for $[M]^+$, 423.27600; Found, 423.27720.

2-(4-(2-decyl-1,3-dioxo-2,3-dihydro-1*H*-benzo[de]isoquinolin-6-yl)piperazin-1-yl)-ethan-1-aminium chloride (4d)

Compound **4d** was prepared in the same manner as for **4a** from **3d** in 69% yield as a yellow solid. ^{1}H-NMR (400 MHz, CD_3OD) δ: 8.56-8.53 (m, 2H), 8.49 (d, 1H, J = 8.3 Hz), 7.84 (t, 1H, J = 8.3 Hz), 7.46 (d, 1H, J = 7.3 Hz), 4.20 (t, 2H, J = 7.3 Hz), 3.90-3.58 (m, 12H), 1.74-1.67 (m, 2H), 1.41-1.30 (m, 14H), 0.90 (t, 3H, J = 7.3 Hz); ^{13}C-NMR (101 MHz, CD_3OD): δ 164.1, 163.7, 153.7, 137.7, 131.8, 130.8, 129.8, 129.3, 128.1, 126.3, 126.0, 124.7, 122.9, 117.8, 115.7, 53.3, 52.5, 49.6, 39.8, 33.9, 33.7, 31.6, 29.4, 29.2, 29.0, 27.6, 27.2, 26.7, 22.3, 13.0; HRMS (ESI$^+$): calcd for $[M]^+$, 465.32295; Found, 465.32149.

2-(4-(2-(2-((*tert*-butoxycarbonyl)amino)ethyl)-1,3-dioxo-2,3-dihydro-1*H*-benzo[de]isoquinolin-6-yl)piperazin-1-yl)ethan-1-aminium chloride (4e)

Compound **4e** was prepared in the same manner as for **4a** from **3e** in 75% yield as a yellow solid. ^{1}H-NMR (400 MHz, CD_3OD) δ: 8.67-8.59 (m, 3H), 7.90 (t, 1H, J = 8.3 Hz), 7.52 (d, 1H, J = 7.3 Hz), 4.49 (t, 2H, J = 7.3 Hz), 3.71-3.57 (m, 12H) ; ^{13}C-NMR (101 MHz, DMSO-d_6): δ 164.0, 163.5, 153.8, 131.8, 130.6, 130.2, 129.1, 126.3, 125.2, 122.8, 115.6, 52.9, 51.4, 49.2, 33.3; HRMS (ESI$^+$): calcd for $[M]^+$, 368.20865; Found, 368.20952.

2-(1-(2-(4-((*tert*-butoxycarbonyl)amino)butyl)-1,3-dioxo-2,3-dihydro-1*H*-benzo[de]isoquinolin-6-yl)-1l3,4-bromazinan-4-yl)ethan-1-aminium chloride (4f)

Compound **4f** was prepared in the same manner as for **4a** from **3f** in 64% yield as a yellow solid. ^{1}H-NMR (400 MHz, CD_3OD) δ: 8.63-8.56 (m, 3H), 7.88 (t, 1H, J = 8.3

Hz), 7.51 (d, 1H, *J* = 7.3 Hz), 4.22 (t, 2H, *J* = 7.3 Hz), 3.74-3.57 (m, 12H), 3.03 (t, 2H, *J* = 7.3 Hz), 1.87-1.75 (m, 4H); ^{13}C-NMR (101 MHz, DMSO-d_6): δ 164.0, 163.5, 154.3, 132.5, 131.3, 130.9, 129.4, 127.0, 125.8, 123.1, 117.2, 116.3, 53.4, 51.9, 49.7, 33.8, 25.2, 25.1; HRMS (ESI$^+$): calcd for $[M]^+$, 396.23995; Found, 396.24139.

***tert*-butyl-(5-((1,3-dioxo-2-pentyl-2,3-dihydro-1*H*-benzo[*de*]isoquinolin-6-yl)-amino)pentyl)carbamate (5a)**

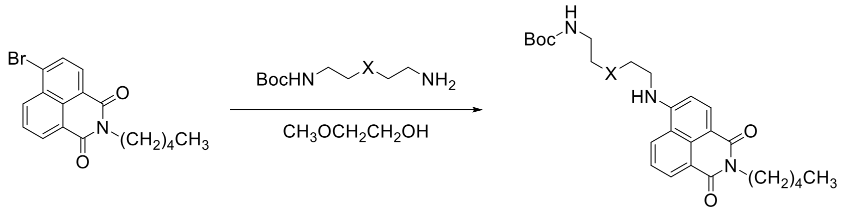

1b **5a, b**

5a (X=CH$_2$)
5b (X=NMe)

To a stirred solution of **1b** (300 mg, 0.86 mmol) in 2-methoxyethanol (40 mL) was added *tert*-butyl-*N*-(5-aminopentyl)carbamate (210 mg, 1.0 mmol, 1.2 eq). The resulting mixture was stirred at 120 °C for 16 h and was evaporated *in vacuo*. The crude product was purified by silica-gel column chromatography with $CHCl_3$ / MeOH (9/1, v/v) eluent to yield 310 mg (77%) of **5a** as a pale yellow oil. ^{1}H-NMR (400 MHz, $CDCl_3$) δ: 8.58 (d, 1H, *J* = 7.2 Hz), 8.46 (d, 1H, *J* = 8.5 Hz), 8.17 (d, 1H, *J* = 7.8 Hz), 7.61 (t, 1H, *J* = 7.8 Hz), 6.70 (d, 1H, *J* = 7.8 Hz), 5.38 (bs, 1H), 4.54 (bs, 1H), 4.15 (t, 2H, *J* = 7.5 Hz), 3.43-3.39 (m,), 3.22-3.10 (m), 1.85 (t), 1.72 (t), 1.59-1.25 (m), 0.90 (t, 3H); ^{13}C-NMR (101 MHz, $CDCl_3$): δ 164.7, 164.1, 156.2, 156.0, 149.5, 134.4, 131.0, 129.8, 126.1, 124.6, 123.1, 120.2, 104.2, 79.0, 43.6, 40.3, 40.1, 30.1, 29.6, 29.3, 27.8, 24.2, 23.8, 22.4, 14.0; ESI-MS: calcd for $[M-H]^-$, 466.2; found, 466.1.

tert-butyl-(2-((2-((1,3-dioxo-2-pentyl-2,3-dihydro-1*H*-benzo[*de*]isoquinolin-6-yl)-amino)ethyl)(methyl)amino)ethyl)carbamate (5b)

Compound **5b** was prepared in the same manner as for **5a** with *tert*-butyl (2-((2-aminoethyl)(methyl)amino)ethyl)carbamate, instead of *tert*-butyl -*N*-(5-aminopentyl)carbamate, in 43% yield as a pale yellow oil. ^{1}H-NMR (400 MHz, $CDCl_3$) δ: 8.58 (d, 1H, *J* = 7.2 Hz), 8.46 (d, 1H, *J* = 8.5 Hz), 8.14 (d, 1H, *J* = 7.8 Hz), 7.63 (t, 1H, *J* = 7.8 Hz), 6.68 (d, 1H, *J* = 7.8 Hz), 6.15 (bs, 1H), 4.79 (bs, 1H), 4.15 (t, 2H, *J* = 7.5 Hz), 3.45-3.39 (m, 2H), 3.32-3.29 (m, 2H), 2.84 (t, 2H, *J* = 7.8 Hz), 2.62 (t, 2H, *J* = 7.8 Hz), 2.34 (s, 3H), 1.74-1.69 (m, 2H), 1.42-1.36 (m, 13H), 0.90 (t, 3H, *J* = 7.5 Hz); ^{13}C-NMR (101 MHz, $CDCl_3$): δ 164.7, 164.1, 156.0, 149.4, 134.4, 131.0. 129.8, 126.2, 124.7, 123.1, 120.4, 110.4, 104.4, 56.8, 55.3, 40.1, 29.3, 28.4, 28.3, 27.9, 22.4, 14.0; ESI-MS: calcd for $[M+Na]^+$, 505.2; found, 505.4.

2-(4-(1,3-dioxo-2-propyl-2,3-dihydro-1*H*-benzo[de]isoquinolin-6-yl)piperazin-1-yl)-ethan-1-aminium chloride (6a)

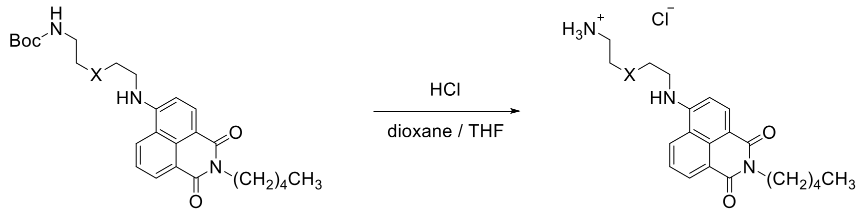

5a, b

5a ($X=CH_2$)
5b (X=NMe)

6a, b

6a ($X=CH_2$)
6b (X=NMe)

Compound **5a** was deprotected in the same manner as for **4a**, to give **6a** in 23% yield as a yellow solid. ^{1}H-NMR (400 MHz, CD_3OD) δ: 8.57-8.52 (m, 2H), 8.38 (d, 1H, *J* = 7.3 Hz), 7.67 (t, 1H, *J* = 8.3 Hz), 6.82 (d, 1H, *J* = 7.3 Hz), 4.12 (t, 2H, *J* = 7.3 Hz), 3.51 (t, 2H, *J* = 7.3 Hz), 2.97 (t, 2H), 1.89-1.85 (m, 2H), 1.79-1.69 (m, 2H), 1.63-1.57 (m, 2H),

1.54-1.46 (m, 2H), 1.41 (m, 4H), 0.95 (t, 3H, J = 7.3 Hz); ^{13}C-NMR (101 MHz, CD_3OD): δ 164.8, 164.4, 151.2, 134.5, 130.8, 129.9, 127.9, 124.1, 122.1, 120.5, 108.0, 103.6, 42.6, 39.6, 39.2, 39.0, 28.9, 27.6, 27.4, 27.0, 26.6, 23.6, 23.0, 22.0, 12.9; HRMS (ESI^+): calcd for $[M]^+$, 368.23380; Found, 368.23392.

2-(4-(1,3-dioxo-2-propyl-2,3-dihydro-1*H*-benzo[de]isoquinolin-6-yl)piperazin-1-yl)-ethan-1-aminium chloride (6b)

Compound **5b** was deprotected in the same manner as for **4a**, to give **6b** in 23% yield as a yellow solid. ^{1}H-NMR (400 MHz, CD_3OD) δ: 8.70 (d, 1H, J = 7.3 Hz), 8.52 (d, 1H, J = 7.3 Hz), 8.40 (d, 1H, J = 7.3 Hz), 7.70 (t, 1H, J = 8.3 Hz), 6.96 (d, 1H, J = 7.3 Hz), 4.12 (t, 2H, J = 7.3 Hz), 4.00 (t, 2H, J = 7.3 Hz), 3.85-3.56 (m, 4H), 3.51 (t, 2H, J = 7.3 Hz), 3.11 (s, 3H), 1.74-1.67 (m, 2H), 1.45-1.38 (m, 4H), 0.95 (t, 3H, J = 7.3 Hz); ^{13}C-NMR (101 MHz, CD_3OD): δ 164.4, 164.0, 149.5, 133.9, 130.7, 129.2, 128.3, 124.5, 121.8, 120.6, 109.9, 104.1, 54.8, 53.0, 39.7, 37.7, 35.2, 34.0, 29.0, 27.4, 22.0, 12.9; HRMS (ESI^+): calcd for $[M]^+$, 383.24470; Found, 383.24468.
